# Prevalences of Parental and Peer Support and Their Independent Associations With Mental Distress and Unhealthy Behaviours in 53 Countries

**DOI:** 10.3389/ijph.2022.1604648

**Published:** 2022-10-10

**Authors:** Lian Li, Guodong Xu, Dongsheng Zhou, Ping Song, Yucheng Wang, Guolin Bian

**Affiliations:** ^1^ Ningbo Kangning Hospital, Ningbo, China; ^2^ Ningbo Medical Centre Li Huili Hospital, Ningbo, China

**Keywords:** adolescents, mental distress, parental support, peer support, unhealthy behaviours

## Abstract

**Objective:** Parental and peer support are both associated with mental distress and unhealthy behaviour indices in adolescents.

**Methods:** We used the Global School-Based Student Health Survey data (*n* = 192,633) from 53 countries and calculated the weighted prevalence of individual and combined parental and peer support. Multiple logistic regression analysis was used to estimate the adjusted associations between combined parental and peer support with mental distress and unhealthy behaviours.

**Results:** The prevalence figures for having all four categories of parental support and two peer-support were 9.7% and 38.4%, respectively. Compared with no parental support, adolescents with all four parental support negatively associated with all five mental distress and eight unhealthy behaviours factors, and the ORs ranged from 0.19 to 0.75. Additionally, adolescents with two peer support were negative association with all mental distress and four health risk behaviours, and positively associated with a sedentary lifestyle.

**Conclusion:** Parental and peer support were lacking in some countries, while greater parental and peer support were negative associated with mental distress and most unhealthy behaviours in adolescents, and the relationships were independent.

## Introduction

Adolescents represent nearly a quarter of the world’s population, and approximately 90% live in low-income and middle-income countries (LMICs). Adolescence is a vital period in life—with changes in physical, cognitive, social, and emotional development [1], and families exert the primary influence on child development [2]. Family support—such as parental respect and attention—is the most important factor that protects against poor health outcomes in adolescents by helping them adopt effective measures to cope with stress and adverse life events [3]. Adolescents might, as a result, drive toward self-involvement and independence, and tend to make friends and rely on them [1]. Therefore, friendships are also very important to the health of adolescents.

Mental distress was shown to be prevalent in adolescents [4], especially in LMICs. Half of mental health distress in adulthood starts by age 14, but most cases go undetected [5]. A study of LMICs showed that the overall pooled prevalence of anxiety in 82 countries was 9.0% (7.0%–12.0%) [6]. Unhealthy behaviours (tobacco, alcohol, and marijuana use, and physical inactivity) contribute to the incidence of non-communicable diseases in adults, and increase the short-term or long-term likelihood of morbidity and mortality [7]. For example, in individuals older than 60 years of age, tobacco use accounted for 10% of disability-adjusted life-years (DALYs), alcohol use for 7%, marijuana use for 2%, and physical inactivity accounted for 7% [8]. Premature sexual activity contributes to epidemics of HPV and HIV, and can result in adolescent pregnancy that affects adolescents’ present and future health, and accounts for 4% of DALYs [8]. The reduction in mental distress and unhealthy behaviours is thus important in improving childhood development.

Previous studies that explored individual parental and peer support all entailed protective factors related to some mental distress and unhealthy behaviours in LMICs [6, 9–11]. Moreover, in some developed countries adolescents who reported more family connections also delayed sexual initiation and exhibited lower levels of substance use [3, 12]. However, investigators have not previously been able to examine the effects of combined parental support (where parents checked homework, understood problems, concerned with adolescent free time, and where parents respected privacy) and peer support (having close friendships and supportive classmates)—all of which concentrates on investigating the relationship between individual parental or peer support and mental health factors.

In order to help shape adolescent mental distress and unhealthy behaviour-prevention strategies, we investigated the associations between a comprehensive panel of parental and peer support with mental distress and unhealthy behaviours using 53 country-representative samples from the GSHS—varying in gender, World Bank country-income classification, and WHO regions.

## Methods

### Data Sources

We used the publicly available data from the GSHS. GSHS is a self-administered, school-based survey jointly developed by the World Health Organization (WHO) and the United States Centers for Disease Control and Prevention (CDC). The details of the survey methodology and questionnaires can be found at the websites of the WHO and CDC (http://www.who.int/chp/gshs and http://www.cdc.gov/gshs, respectively). The primary aim of the GSHS was to assess and quantify the risks and protective factors involved in major non-communicable diseases—including alcohol, tobacco, and drug use, hygienic practices, sexual behaviours, mental health, violence, and unintentional injury [13]. The survey uses a standardized two-stage probability sampling design for the selection process within each participating country. In the first stage, schools were randomly sampled from all schools in the country according to probability proportional to size sampling. In the second stage, classes with targeted-age students in each selected school were randomly sampled from systematic equal-probability sampling. All students in the selected class were then ultimately eligible to participate in the survey [13], and all data collection was performed during regular class time. The questionnaire was translated into the local language of each country and finished by students using a self-report, computer-scannable form. All GSHS surveys were approved by the Ministry of Health or Education and Ethics Committee, and informed consent was received from students, parents, and/or school officials, where necessary.

We selected nationally representative datasets that included all parental and peer-support variables. The GSHS entailed three historical surveys, such that if a particular country already had two or more datasets, we chose the most recent dataset. Finally, a total of 53 countries with the survey conducted between 2009 and 2015 were included in the current study. Our study populations restricted to in-school adolescents for aged 13–17 years. The corresponding country-income levels of the included countries were also obtained based on their World Bank classifications at the time the survey was conducted [14]. The detailed characteristics of included countries are listed in Table 1.

**TABLE 1:** Survey characteristics (Low-income and middle-income countries, 2009–2015).

	Survey (year)	n/N	Response rate (%)	Boys (%)	Country income level
Africa Region					
Benin	2009	2679/2690	99.59	65.39	Low
Mauritania	2010	2013/2063	97.58	46.76	Lower middle
Mozambique	2015	1833/1918	95.57	52.74	Low
Namibia	2013	4436/4531	97.90	46.75	Upper middle
Seychelles	2015	2452/2540	96.54	44.53	High
Swaziland	2013	3645/3680	99.05	47.28	Lower middle
United Republic of Tanzania	2014	3745/3793	98.73	48.03	Low
Eastern Mediterranean Region					
Afghanistan	2014	2459/2579	95.35	42.78	Low
Egypt	2011	2471/2568	96.22	46.10	Lower middle
Iraq	2012	1998/2038	98.04	56.51	Upper middle
Kuwait	2015	3391/3637	93.24	47.80	High
Lebanon	2011	2257/2286	98.73	46.24	Upper middle
Morocco	2010	2890/2924	98.84	52.51	Lower middle
Oman	2015	3392/3468	97.81	46.51	High
Qatar	2011	1814/2021	89.76	42.19	High
Syrian Arab Republic	2010	3073/3102	99.07	39.54	Lower middle
United Arab Emirates	2010	2528/2581	97.95	41.42	High
America Region					
Antigua and Barbuda	2009	1260/1266	99.53	46.33	Upper middle
Argentina	2012	27579/28368	97.22	47.17	Upper middle
Barbados	2011	1584/1629	97.24	44.62	High
Belize	2011	2084/2112	98.67	46.70	Lower middle
Bolivia	2012	3665/3696	99.16	50.88	Lower middle
British Virgin Islands	2009	1632/1664	98.08	44.47	High
Bahamas	2013	1333/1357	98.23	44.57	High
Costa Rica	2009	2653/2679	99.03	47.80	Upper middle
Curaçao	2015	2732/2765	98.81	43.82	High
El Salvador	2013	1872/1915	97.75	53.82	Lower middle
Honduras	2012	1750/1779	98.37	48.18	Lower middle
Jamaica	2010	1581/1623	97.41	47.68	Upper middle
Peru	2010	2869/2882	99.55	48.55	Upper middle
Saint Kitts and Nevis	2011	1726/1740	99.20	42.60	High
Suriname	2009	1665/1698	98.06	50.56	Upper middle
Trinidad and Tobago	2011	2783/2811	99.00	53.73	High
Western Pacific Region					
Brunei Darussalam	2014	2582/2599	99.35	46.24	High
Cook Islands	2015	695/701	99.14	49.13	NA
Kiribati	2011	1561/1582	98.67	43.24	Lower middle
Lao People’s Democratic Republic	2015	3662/3683	99.43	45.64	Lower middle
Malaysia	2012	25369/25507	99.46	49.77	Upper middle
Mongolia	2013	5363/5393	99.44	46.82	Lower middle
Philippines	2015	8707/8761	99.38	45.31	Lower middle
Samoa	2011	2150/2418	88.92	38.03	Lower middle
Solomon Islands	2011	1400/1421	98.52	52.31	Lower middle
Tokelau	2014	136/140	97.14	47.58	NA
Tonga	2010	2154/2211	97.42	45.84	Lower middle
Tuvalu	2013	911/943	96.61	47.23	Upper middle
Vanuatu	2011	867/1119	77.48	44.34	Lower middle
Vietnam	2013	3311/3331	99.40	46.68	Lower middle
Wallis and Futuna	2015	1090/1117	97.58	44.96	NA
South-East Asia Region					
Bangladesh	2014	2957/2989	98.93	39.88	Lower middle
Indonesia	2015	11071/11142	99.36	45.42	Lower middle
Maldives	2014	3385/3493	96.91	40.82	Lower middle
Thailand	2015	5802/5894	98.44	41.37	Upper middle
Timor-Leste	2015	3616/3704	97.62	46.00	Lower middle
Total	—	192633/196551	98.00	46.99	—

NA: not available.

## Measures

### Parental and Peer Support

Parental support was assessed by four components (parents checked homework, parents understood problems, parents concerned free time, and parents respected for privacy). “Parents checked homework” was examined with the question “Percentage of students whose parents or guardians check to see if your homework was done most of the time or always during the past 30 days?” with a binary response of “yes” or “no”. “Parents understood problems” was examined with the question “Percentage of students whose parents or guardians understand your problems and worry most of the time or always during the past 30 days?” with a binary response of “yes” or “no”. “Parents concerned free time” was examined with the question “Percentage of students whose parents or guardians really know what you were doing with your free time most of the time or always during the past 30 days?” with a binary response of “yes” or “no.” “Parental respect for privacy” was examined with the question, “Percentage of students whose parents or guardians go through your things without your approval never or rarely during the past 30 days?” with a binary response of “yes” or “no.” Peer support was assessed by two components (close friendships and supportive classmates). “Close friendships” was examined with the question “Percentage of students who had no close friends?” with a binary response of “yes” or “no”. “Supportive classmates” was examined with the question “Percentage of students who reported most of the students in your school as kind and helpful most of the time or always during the past 30 days?” with a binary response of “yes” or “no” (Supplementary Table S1). An individual answering “yes” meant a question score of 1; otherwise, the score was 0—except for the question of close friendships, where the answer “yes” meant a score of 0. The combined parental and peer support of the young adolescents was calculated by summing the aforementioned four parental support question scores and the two peer support question scores.

### Mental Health Factors

We also selected five mental health outcome variables to evaluate their associations with parental and peer support. These were loneliness (yes, no), insomnia due to anxiety (yes, no), suicidal ideation (yes, no), suicidal planning (yes, no), and suicide attempts (yes, no). The completed questions regarding mental health factors, their answers, and coding are presented in Supplementary Table S1.

### Health Risk Behaviours

We also selected eight health-risk behaviour outcome variables to evaluate their associations with parental and peer support. These were violence (yes, no), hygienic practices (yes, no), premature sexual activities (yes, no), current tobacco use (yes, no), current alcohol use (yes, no), current marijuana use (yes, no), sedentary lifestyle (yes, no), and school truancy (yes, no). The completed questions regarding health-risk behaviours, their answers, and coding are presented in Supplementary Table S1.

### Confunding Factors

We also selected age, sex, BMI and hunger status as confunding factors. Hunger status was examined with the question “Percentage of students who went hungry most of the time or always because there was not enough food in your home during the past 30 days?” with a binary response of “yes” or “no,” and hunger status was defined as socioeconomic status of adolescents family.

## Statistical Analyses

The frequencies of individual and combined parental and peer support were based on individual data from each country survey, and we calculated the overall prevalence of individual and combined parental and peer support for all participants. As the GSHS uses a complex sampling design, data analyses should take this into account. We thus calculated the weighted prevalence estimates and corresponding 95% CIs using the SURVEYMEANS procedure in SAS (version 9.4). We added weights, stratum, and a primary sampling unit (PSU) to every school-attending child to reflect the weighting process and the two-stage sampling design. The weighting allowed the results to be generalized to the study population and the national student population, the stratum reflected the first stage of the GSHS sampling (at the school level), and the PSU reflected the second stage (the classroom level).

Pooled regional and country-income levels and overall estimates were calculated by random-effects meta-analysis using STATA (version 12.0), and we used the I^2^ statistic to estimate heterogeneity. Subgroup analyses were stratified by gender (boys vs. girls). We used logistic regression models to analyse the relationships between combined parental and peer support and mental health factors and health risk behaviours, adjusting for age, sex, BMI, and food insecurity. The food insecurity variable was used as a proxy for socioeconomic status (SES) [15]. *p* values <0.05 were considered statistically significant. SAS version 9.4 (SAS Institute, Cary, NC, United States) and STATA version 12.0 (Stata Corporation; College Station, TX, United States) were used to perform statistical analyses.

## Results

Table 1 depicts the characteristics of the survey and participants from the included GSHS datasets. Ultimately, fifty-three countries or regions were included from five WHO regions: 7 from the African Region; 10 from the Eastern Mediterranean Region; 16 from the Region of the Americas; 15 from the Western Pacific Region; and 5 from the South-East Asia Region (Supplementary Figure S1). According to the World Bank country-income classification based upon the years of the survey, included surveys were categorized into 4 low-income countries, 22 lower middle-income countries, 12 upper middle-income countries, 12 high-income countries, and 3 countries or regions with no classification information in the World Bank. This distribution corresponded to a total of 192,633 young adolescents who attended school (46.99% boys and 53.01% girls) in our analysis. The median sample size for each country was 2471 (1665–3391), and the overall response rate was 98.00% (range, 77.48%–99.57%).

**FIGURE 1:**
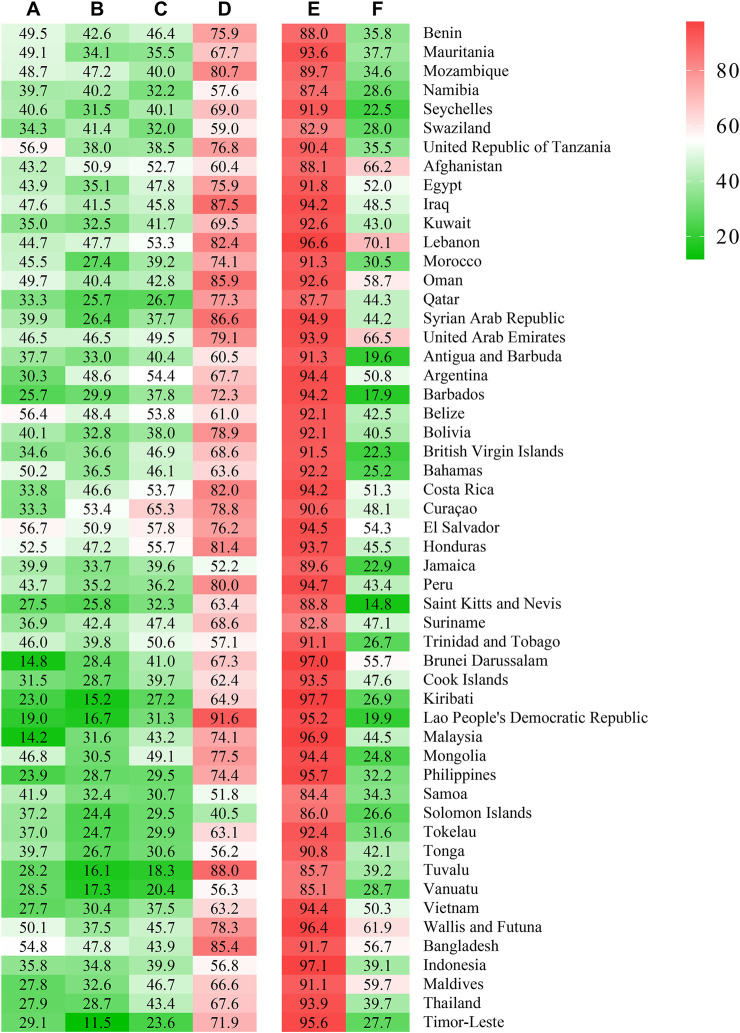
Individual parental and peer support in adolescents across country (Low-income and middle-income countries, 2009–2015). **(A)**: Parents checked homework; **(B)**: Parents understood problems; **(C)**: Parents concerned free time; **(D)**: Parents respected for privacy; **(E)**: Having close friendships; **(F)**: Having supportive classmates.

The overall prevalence of the categories parents checked homework, parents understood problems, parents concerned free time, and parents respected for privacy was 39.6%, 35.4%, 40.8%, and 71.4%, respectively (Table 2; Supplementary Table S2). There were 92.6% of school-attending adolescents who reported having one or more close friends and 40.5% who reported having supportive classmates (Table 2; Supplementary Table S2). Girls more frequently stated that parents understood problems and parents concerned free time than did boys (36.2% vs. 34.3% and 41.6% vs. 38.2%, respectively) (Table 2; Supplementary Tables S3, S4), and girls reported having slightly more supportive classmates compared with boys (41.4% vs. 37.6%). Low socioeconomic status more frequently stated low parental and peer support (Supplementary Tables S5, S6). The adolescents from the Western Pacific Region exhibited the lowest individual parental support (Table 2; Supplementary Table S2), and adolescents from the Africa Region reported the lowest prevalence in possessing close friendships and supportive classmates. According to the World Bank country-income classification, adolescents from low-income countries manifested the highest prevalence of individual parental support, while they showed the highest prevalence of having no close friendships (Table 2). At the country level, the overall prevalence of parents checking homework was highest in the United Republic of Tanzania (56.9%) and lowest in Malaysia (14.2%). The highest overall prevalence of parents understanding students problems and concerned students free time was in Curaçao (53.4% and 65.3%), with the lowest in Timor-Leste (11.5%) and Tuvalu (18.3%). The overall prevalence of parents respected for privacy was highest in the Lao People’s Democratic Republic (91.6%), and lowest in the Solomon Islands (40.5%). The adolescents from Kiribati reported the highest prevalence of having one or more close friendships (97.7%), while Suriname exhibited the lowest (82.8%). The adolescents from Lebanon reported the highest prevalence of having supportive classmates (70.1%), while this index was lowest in Saint Kitts and Nevis (14.8%) (Supplementary Table S2; Figure 1).

**TABLE 2:** The prevalence of individual parental and peer support among young in-school adolescents by region, country income level, and gender groups (Low-income and middle-income countries, 2009–2015).

	Parental support			Peer support		
	Parents checked homework	Parents understood problems	Parents concerned free time	Parents respected for privacy	Having close friendships	Having supportive classmates
Region						
Africa Region	45.5 (39.1–51.9)	39.0 (35.5–42.6)	37.7 (33.7–41.6)	69.5 (63.1–75.9)	89.1 (86.3–92.0)	31.6 (27.1–36.2)
Eastern Mediterranean Region	43.0 (39.3–46.7)	37.2 (31.5–42.9)	43.6 (38.2–49.0)	78.1 (73.7–82.5)	92.6 (91.2–94.0)	52.4 (44.8–60.2)
America Region	41.6 (36.4–46.7)	41.4 (37.1–45.6)	48.4 (43.2–53.6)	70.2 (65.8–74.6)	92.2 (91.0–93.3)	38.3 (31.2–45.4)
Western Pacific Region	30.6 (23.1–38.1)	26.9 (23.0–30.8)	35.0 (30.3–39.8)	66.2 (59.7–72.8)	93.7 (92.4–95.0)	38.1 (31.0–45.3)
South-East Asia Region	34.7 (28.1–41.3)	30.9 (20.7–41.2)	39.5 (31.1–47.8)	69.6 (61.4–77.8)	94.0 (91.7–96.2)	44.6 (32.2–57.0)
Country income level						
Low	50.1 (44.7–55.6)	43.9 (38.8–48.9)	44.0 (38.3–49.6)	73.6 (67.2–79.9)	89.3 (87.9–90.6)	42.4 (32.7–52.0)
Lower middle	38.8 (34.3–43.2)	31.4 (27.0–35.9)	38.0 (33.7–42.2)	69.1 (64.3–74.0)	92.4 (91.0–93.7)	38.3 (33.3–43.4)
Upper middle	36.0 (27.7–44.3)	39.1 (34.5–43.6)	44.5 (39.9–49.1)	71.1 (66.0–76.2)	92.6 (90.9–94.3)	42.4 (34.7–50.0)
High	37.5 (28.4–46.5)	36.4 (30.8–42.1)	44.2 (37.2–51.1)	72.0 (66.7–77.4)	92.4 (90.7–94.2)	40.9 (29.2–52.5)
Gender						
Boys	39.8 (35.1–44.4)	34.3 (28.4–40.3)	38.2 (33.7–42.7)	71.0 (67.3–74.6)	92.5 (91.6–93.5)	37.6 (32.2–42.9)
Girls	38.6 (32.7–44.5)	36.2 (30.0–42.5)	41.6 (36.1–47.1)	70.1 (63.8–76.3)	92.7 (91.1–94.3)	41.4 (32.5–50.2)
Total	39.6 (34.9–44.3)	35.4 (29.3–41.4)	40.8 (35.9–45.7)	71.4 (66.2–76.6)	92.6 (91.4–93.8)	40.5 (32.9–48.1)

**FIGURE 2:**
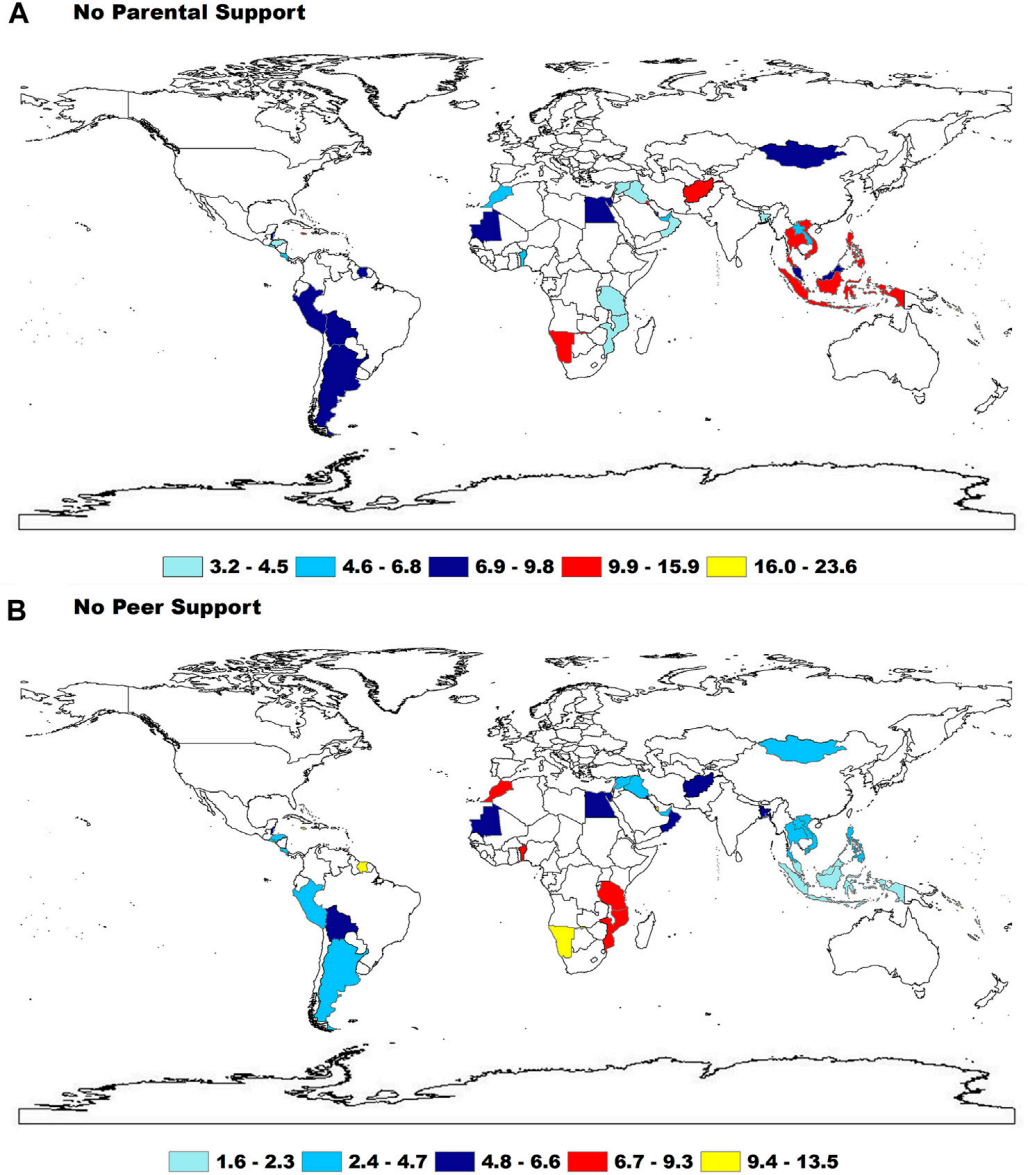
The prevalence of having no any parental and peer support among adolescents by WHO regions (Low-income and middle-income countries, 2009–2015).

The overall prevalence of having no parental supportive was 8.8%, with the lowest in the Eastern Mediterranean Region (6.4%) and the highest in the Western Pacific Region (12.5%); with respect to country, it was lowest in Iraq (3.2%) and highest in Vanuatu (23.6%). The prevalence of having all four categories of parental support was 9.7%, with the lowest in Western Pacific Region (5.3%) and the highest in the Eastern Mediterranean Region (14.0%); with respect to country, the lowest was in Vanuatu (1.3%) and the highest in El Salvador (24.9%). The prevalence of having no peer support was 5.3% overall, with the lowest in the South-East Asia Region (4.1%) and the highest in the Africa Region (8.2%): for countries, the lowest was in Kiribati (1.6%) and the highest was in Swaziland (13.5%). The prevalence of having two peer-support mechanisms was 38.4%, with the lowest in the Africa Region (29.0%) and the highest in the Eastern Mediterranean Region (49.9%): for countries, the lowest was in Barbados (17.2%) and the highest was in Lebanon (68.7%) (Figure 2; Supplementary Tables S7, S8).

Overall, regarding the proxy of mental health factors, loneliness, insomnia due to anxiety, suicidal ideation, suicidal planning, and suicidal attempts were associated with parental support, and the associations were significantly enhanced by the increased number of parental support, as demonstrated by the significant p_trend_ value. Compared with adolescents who received no parental support, adolescents who received support in all four parental categories had the lowest ORs, with the ORs ranging from 0.30 (0.28–0.32) for suicidal ideation to 0.51 (0.47–0.56) for insomnia due to anxiety. Also, all mental health factors were associated with peer support. Additionally, the associations were significantly enhanced commensurate with increased numbers of parental support mechanisms, except for insomnia due to anxiety. The ORs of adolescents who had two peer support ranged from 0.36 (0.34–0.39) for suicidal attempts to 0.64 (0.59–0.69) for insomnia due to anxiety (Table 3). In addition, boys and girls had no difference in relationships between parental and peer support and mental health factors (Supplementary Tables S9, S10).

**TABLE 3:** The relationship between the number of parental and peer support and mental distress (Low-income and middle-income countries, 2009–2015).

	Loneliness	Insomnia due to anxiety	Suicidal ideation	Suicidal plan	Suicidal attempt
Number of parental support					
0	1 [Reference]	1 [Reference]	1 [Reference]	1 [Reference]	1 [Reference]
1	0.75 (0.71–0.79)	0.73 (0.69–0.77)	0.67 (0.64–0.70)	0.72 (0.69–0.76)	0.68 (0.64–0.71)
2	0.66 (0.63–0.70)	0.66 (0.62–0.70)	0.49 (0.47–0.52)	0.56 (0.53–0.59)	0.52 (0.49–0.55)
3	0.53 (0.50–0.56)	0.59 (0.55–0.63)	0.41 (0.39–0.44)	0.47 (0.44–0.50)	0.45 (0.42–0.47)
4	0.45 (0.41–0.49)	0.51 (0.47–0.56)	0.30 (0.28–0.32)	0.38 (0.35–0.41)	0.34 (0.32–0.37)
P-trend	<0.001	<0.001	<0.001	<0.001	<0.001
Number of peer support					
0	1 [Reference]	1 [Reference]	1 [Reference]	1 [Reference]	1 [Reference]
1	0.47 (0.44–0.50)	0.64 (0.59–0.69)	0.53 (0.50–0.56)	0.51 (0.48–0.55)	0.48 (0.45–0.51)
2	0.42 (0.38–0.44)	0.64 (0.59–0.69)	0.42 (0.39–0.45)	0.40 (0.38–0.43)	0.36 (0.34–0.39)
P-trend	<0.001	1.000	<0.001	<0.001	<0.001

Multivariable logistic regression model included age, sex, BMI, food insecurity, parental support and peer support.

In terms of health risk behaviours—violence, hygienic practices, premature sexual activities, tobacco use, alcohol use, marijuana use, sedentary lifestyle, and school truancy were all associated with parental support, and the associations were significantly enhanced with increasing number of parental support. Adolescents who received all four parental support had the lowest ORs, with the ORs ranging from 0.19 (0.16–0.24) for marijuana use to 0.75 (0.63–0.88) for premature sexual activity. Parental support mechanisms were strongly associated with tobacco use, marijuana use, and school truancy. Moreover, five of eight health risk behaviours (violence, premature sexual activity, tobacco use, marijuana use, and sedentary lifestyle) were associated with peer support, and the association was significantly enhanced with an increased number of peer support. For sedentary lifestyles, the OR was 1.35 (1.27–1.42) in adolescents who received both peer-support mechanisms, while the ORs for the other four health risk behaviours were all approximately 0.60 (Table 4). Boys and girls also had no difference in relationships between parental and peer support and health risk behaviours (Supplementary Tables S9, S10).

**TABLE 4:** The relationship between the number of parental and peer support and health risk behaviours (Low-income and middle-income countries, 2009–2015).

	Violence	Hygiene practices	Premature sexual	Tobacco use	Alcohol use	Marijuana use	Sedentary	School truancy
Number of parental support								
0	1 [Reference]	1 [Reference]	1 [Reference]	1 [Reference]	1 [Reference]	1 [Reference]	1 [Reference]	1 [Reference]
1	0.68 (0.65–0.70)	1.03 (0.99–1.07)	1.00 (0.89–1.12)	0.71 (0.66–0.75)	0.73 (0.70–0.77)	0.61 (0.59–0.67)	0.85(0.82–0.89)	0.78 (0.75–0.81)
2	0.58 (0.56–0.61)	0.89 (0.85–0.92)	0.79 (0.70–0.89)	0.54 (0.50–0.58)	0.63 (0.60–0.67)	0.46 (0.42–0.51)	0.91 (0.87–0.94)	0.62 (0.60–0.65)
3	0.50 (0.48–0.52)	0.76 (0.73–0.79)	0.78 (0.68–0.90)	0.36 (0.33–0.39)	0.60 (0.57–0.63)	0.38 (0.34–0.43)	0.83 (0.79–0.86)	0.48 (0.46–0.50)
4	0.41 (0.39–0.43)	0.71 (0.67–0.74)	0.75 (0.63–0.88)	0.24 (0.21–0.27)	0.48 (0.45–0.51)	0.19 (0.16–0.24)	0.61 (0.58–0.64)	0.36 (0.34–0.39)
P-trend	<0.001	<0.001	<0.001	<0.001	<0.001	<0.001	<0.001	<0.001
Number of peer support								
0	1 [Reference]	1 [Reference]	1 [Reference]	1 [Reference]	1 [Reference]	1 [Reference]	1 [Reference]	1 [Reference]
1	0.81 (0.77–0.86)	1.08 (1.03–1.14)	0.72 (0.62–0.85)	0.66 (0.61–0.72)	0.99 (0.92–1.06)	0.62 (0.55–0.70)	1.12 (1.06–1.19)	1.03 (0.98–1.09)
2	0.63 (0.59–0.66)	0.92 (0.87–1.04)	0.57 (0.48–0.67)	0.55 (0.50–0.60)	0.96 (0.89–1.03)	0.55 (0.49–0.63)	1.35 (1.27–1.42)	0.95 (0.90–1.00)
P-trend	<0.001	<0.001	<0.001	<0.001	0.026	<0.001	<0.001	<0.001

Multivariable logistic regression model included age, sex, BMI, food insecurity, parental support and peer support.

## Discussion

From this multi-country study based on nationally representative school-attending adolescents, only 40% of adolescents reported that parents checked homework, parents understood problems, and parents concerned free time—and that they had supportive classmates, while nearly 7 of 10 adolescents reported having parents who respected their privacy, and 9 of 10 adolescents reported having close friendships. Overall, the prevalences of having no parental support vs. having all four categories of parental support were 8.8% and 9.7%, respectively. The prevalence figures for having no vs. both peer-support mechanisms were 5.3% and 38.4%, respectively. Parental support was strongly associated with a lower odds of manifesting mental health factors and health risk behaviours independent of peer support, and adolescents demonstrating higher numbers of individual parental support exhibited stronger associations. Peer support was also strongly associated with mental health factors and major health risk behaviours independent of peer support, and the association was significantly enhanced commensurate with increasing numbers of individual peer support.

With this global study, we reported the prevalence of individual and combined parental and peer support carry substantial variations across countries and regions. At the regional level, adolescents from the Western Pacific Region and South-East Asia Region had the lowest parental support, and the Africa Region reported the lowest peer support. Parenting styles played the pivotal factor in the well-being of the adolescents [16–18]. We assumed that the variations in parenting styles may in part be due to the large differences across diverse economic, cultural, and religious beliefs globally [17]. In fact, parental involvement and support were vital behaviours reflecting parenting style and were usually related to the local socio-political system [18, 19]. In general, the parents who belonged to socially conservative or undemocratic countries were inclined to show more controlling parenting patterns than those in democratic and liberal political systems [18]. In addition, in most Southeast Asian countries, adolescents were used to being asked to respect their elders from childhood on due to Confucian ethics [19], and this relationship might be one reason for the lower parental support. Educational level and socioeconomic status of the parents might also be related to parental support: a family with higher levels of education and socioeconomic status would usually be more inclined to communicate openly with their children and to help their children understand the effects of behaviours and improve their self-efficacy [20]. In addition, the lowest peer support in the Africa Region might be due to poverty, political instability, and social unrest [21].

Mental health factors were all shown to clearly align with parental and peer support, with the associations being independent, and in our study, suicidal behaviours exhibited the strongest association. Previous investigators also described individual parental and peer-support associations with mental health problems (e.g., loneliness, insomnia due to anxiety, suicidal behaviours) in LMIC adolescents [9, 10]. In addition, the Adolescent Brain Cognitive Development (ABCD) study in the United States showed that adolescents aged 9–10 years with lower parental monitoring were more likely to manifest suicidal ideation and to attempt suicide [22]. The French iShare cohort also revealed that those participants who lacked perceived parental support in childhood and adolescence were more likely to exhibit occasional or frequent suicidal thoughts [23]. Adolescence is a vital period of human development due to the physical and psychological changes that take place and the accompanying establishment of self-identity, and, simultaneously, it is also a vulnerable period because of the need to deal with new interpersonal relationships independently [1]. Parental support mechanisms constitute the primary component of positive parent-adolescent relationships, and can confer resilience in combating suicidal behaviours by attenuating the impacts of the risk factors—such as peer victimization and bullying, and feelings of depression, loneliness, and hopelessness [24]. The perception of being cared for, having one’s privacy respected, and parental involvement in the education and lives of adolescents are associated with less adolescent mental distress [19]. Notably, parental support to adolescents can help them manage stress better and keep a healthy physical and mental status [9]. Also, peer support demonstrates a strong independent association with mental disorders by offering positive effects such as increased self-esteem [25], increased resilience [26], increased competence in solving problems, and improved physical and mental health. In our study, mental health factors were all associated with an increase in the number of parental and peer support mechanisms; i.e., those adolescents with a greater number of parental and peer support were less likely to manifest mental health concerns. The findings of our study may therefore present important public health implications in lowering the increasing incidence of mental distress in adolescents. It is vital for parents to realize the importance of parental and peer support to adolescent mental health, to encourage parents to pay more attention to care surrounding parent-adolescent relationships, and to encourage their children to cultivate more friendships.

Common health risk behaviours all showed a clear gradient that aligned with the increasing number of parental support, especially in the use of tobacco and marijuana. Substance use in adolescents exerts great harm on adolescent physical and mental health and also increases the risk for many diseases, such as respiratory illness and digestive system disease. Parents thus play a vital role in the development of health risk behaviours in adolescents. Our results corroborate previous research that concluded that parental monitoring exerts an effective positive influence on smoking intentions and willingness [27]. The observations from the Three Diverse Island Nations also show that lower rates of parental monitoring were significantly associated with more adolescent tobacco use [28]. Therefore, it is important for governments and parents to raise awareness on the effects of increasing parental support so as to decrease adolescent substance use and other health risk behaviours. In our study, we also noted a lack of any significant association between peer support and hygienic practices, alcohol use, or school truancy. Hygienic health practices are related to parental example and instruction, as adolescents principally imitate the behaviour of their parents [29]. Interestingly, those adolescents with a greater number of peer support exhibited risk factors for sedentary lifestyle. Indeed, sedentation is now very popular among adolescents from LMICs [30]. Adolescents spend more time watching TV and playing computer games, which entail long periods of sitting, and they are more likely to conduct these activities with their close friends [30]. Therefore, adolescents with more peer support exhibit more sedentary behaviour. In fact, parental and peer support were highly effective in diminishing health risk behaviours of adolescents, suggesting that parents pay more attention to parent-adolescent relationships.

There were several limitations to the study. First, the data in the GSHS was from school-attending, self-reported information, and recall bias can be problematic. In addition, the study’s validity might also be affected by the children’s ability to understand the questions. Second, due to the loss of some important question variables and high levels of heterogeneity across countries, the possibility of biases in overall prevalence estimates was inevitable. Third, this is a cross-sectional study, and the associations between parental and peer support and mental health factors and health risk behaviours should be interpreted with caution with respect to feasible reverse causation. Finally, in the multivariate logistic regression model, we only included age, sex, BMI, food insecurity, parental support, and peer support—with other confounding factors potentially lost in some countries.

### Conclusion

In conclusion, in our study we demonstrated that there were important differences in the prevalence estimates of individual and combined parental and peer support across countries, regions, and country-income classifications. We have also shown that greater parental and peer support for adolescents was associated with mental health factors and components of the health risk behaviours, and the associations increased with increased parental and peer support. We therefore recommend that policy makers and parents adapt to the importance of parental and peer support to adolescent health, and create and promulgate global and national policies and take related actions to improve both parent-adolescent and peer relationships. Parents and teachers should participate in psychological education programs to aware the important of parental and peer support for reducing mental health problems and increaing the health behaviors, and them should promote a healthy home and school environment for adolescents together.

## Data Availability

Data from this study will be availabled on https://extranet.who.int/ncdsmicrodata/index.php/catalog/GSHS and http://www.cdc.gov/gshs.
