# Supplementary material for: Prevalences of Parental and Peer Support and Their Independent Associations With Mental Distress and Unhealthy Behaviours in 53 Countries
Source: Int J Public Health. 2022 Oct 10;67:1604648. doi: 10.3389/ijph.2022.1604648 (PMC9588916; doi:10.3389/ijph.2022.1604648)
Supplement: Supplementary file 1 [file DataSheet1.docx]

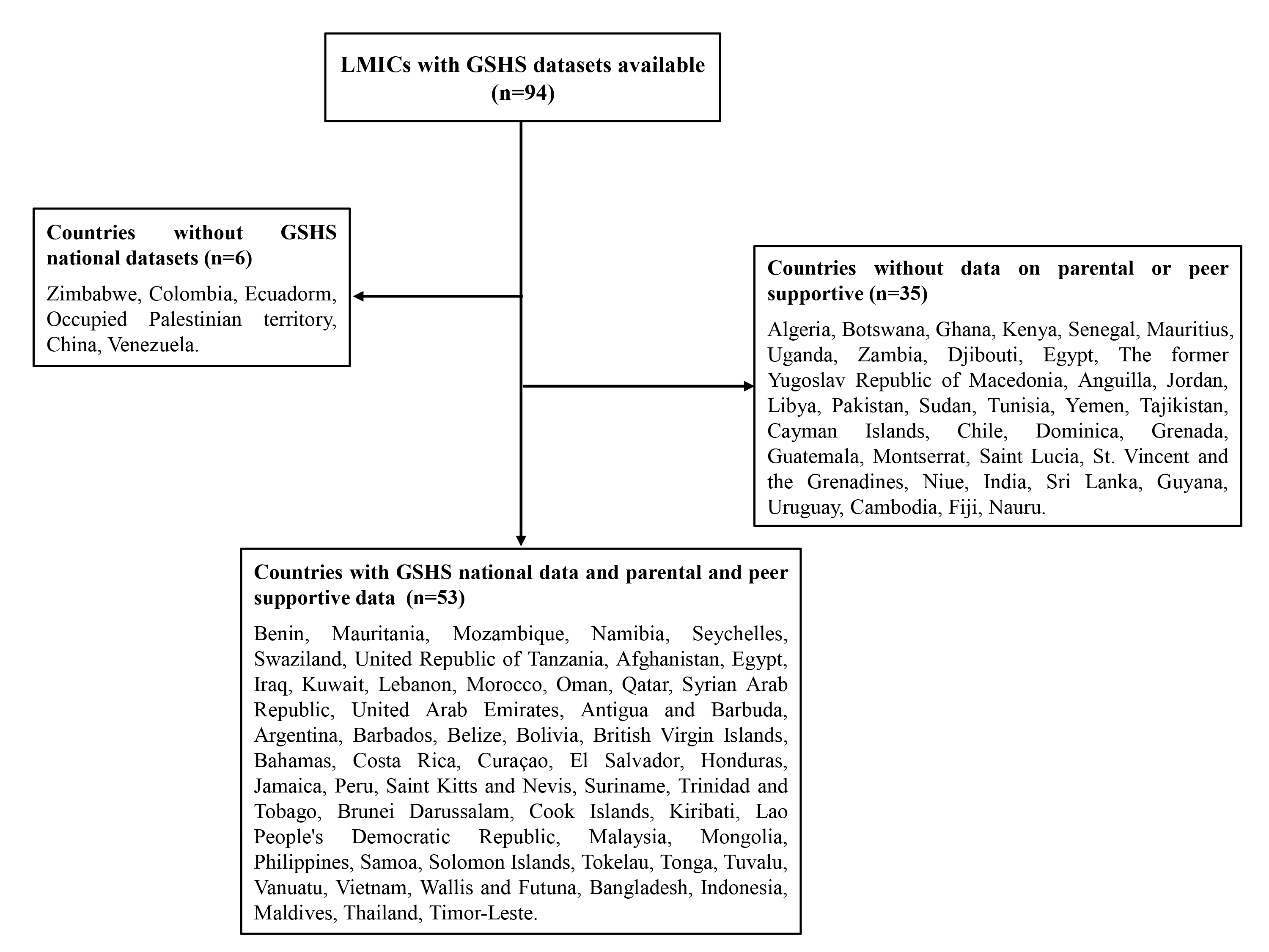


**Supplementary Fig. 1** Selection process for LMICs using GSHS national data (Low-income and middle-income countries, 2009–2015).

**Supplementary Table 1**

The description of adolescent parental and peer support, mental health factors and health risk behaviors in Global School-based Student Health Survey (Low-income and middle-income countries, 2009–2015).

| **Variables** | **Question** | **Answers (Coding)** |
| --- | --- | --- |
| Age | “How old are you?” | 1=11 years old or younger,2 to 5=12 to 15 years old,6=16 years old or older (code as 1 to 6) |
| Sex | “What is your sex?” | 1=Male,2=Female (code as 1 and 2) |
| Hunger (SES) | “Percentage of students who went hungry most of the time or always because there was not enough food in your home during the past 30 days?” | 1=Yes,2=No (code as 1 and 0) |
| **Parental support** | | |
| Parents checked homework | “Percentage of students whose parents or guardians check to see if your homework was done most of the time or always during the past 30 days?” | 1=Yes,2=No (code as 1 and 0) |
| Parents understood problems | “Percentage of students whose parents or guardians understand your problems and worries most of the time or always during the past 30 days?” | 1=Yes,2=No (code as 1 and 0) |
| Parents concerned free time | “Percentage of students whose parents or guardians really know what you were doing with your free time most of the time or always during the past 30 days?” | 1=Yes,2=No (code as 1 and 0) |
| Parents respected for privacy | “Percentage of students whose parents or guardians go through your things without your approval never or rarely during the past 30 days?” | 1=Yes,2=No (code as 1 and 0) |
| **Peer support** | | |
| Close friendships | “Percentage of students who had no close friends?” | 1=Yes,2=No (code as 0 and 1) |
| Supportive classmates | “Percentage of students who reported most of the students in your school kind and helpful most of the time or always during the past 30 days?” | 1=Yes,2=No (code as 1 and 0) |
| **Mental health factors** | | |
| Insomnia due to anxiety | “Percentage of students who most of the time or always were so worried about something that you could not sleep at night during the past 12 months?” | 1=Yes,2=No (code as 1 and 0) |
| Loneliness | “Percentage of students who most of the time or always felt lonely during the past 12 months?” | 1=Yes,2=No (code as 1 and 0) |
| Suicide ideation | “Percentage of students who ever seriously considered attempting suicide during the past 12 months?” | 1=Yes,2=No (code as 1 and 0) |
| Suicide plan | “Percentage of students who make a plan about how they would attempt suicide during the past 12 months?” | 1=Yes,2=No (code as 1 and 0) |
| Suicide attempt | “Percentage of students who actually attempted suicide one or more times during the past 12 months?” | 1=Yes,2=No (code as 1 and 0) |
| **Health risk behaviours** | | |
| Violence | “Percentage of students who were physically attacked or in a physical fight one or more times during the past 12 months or were bullied on one or more days during the past 30 days?” | 1=Yes,2=No (code as 1 and 0) |
| Hygiene practices | “Percentage of students who usually cleaned or brushed their teeth less than one or more times or never or rarely washed their hands before eating and using the toilet during the past 30 days?” | 1=Yes,2=No (code as 1 and 0) |
| Premature sexual | “Percentage of students who ever had sexual intercourse?” | 1=Yes,2=No (code as 1 and 0) |
| Current tobacco use | “Percentage of students who used any tobacco products other than cigarettes, on one or more days during the past 30 days?” | 1=Yes,2=No (code as 1 and 0) |
| Current alcohol use | “Percentage of students who drank at least on drink containing alcohol on one or more of the past 30 days?” | 1=Yes,2=No (code as 1 and 0) |
| Current Marijuana  use | “Percentage of students who used marijuana one or more times during the past 30 days?” | 1=Yes,2=No (code as 1 and 0) |
| Sedentary | “Percentage of students who spent three or more hours per day during a typical or usual day doing sitting activities” | 1=Yes,2=No (code as 1 and 0) |
| School truancy | “Percentage of students who missed classes or school without permission on one or more of the past 30 days” | 1=Yes,2=No (code as 1 and 0) |

**Supplementary Table 2**

The prevalence of individual parental and peer support among in-school adolescents by region, and country groups (Low-income and middle-income countries, 2009–2015).

|  | **Parental support** | | | | **Peer support** | |
| --- | --- | --- | --- | --- | --- | --- |
|  | **Parents checked homework** | **Parents understood problems** | **Parents concerned free time** | **Parents respected for privacy** | **Having close friendships** | **Having supportive classmates** |
| **Africa Region** | | | | | | |
| Benin | 49.5(46.3-52.8) | 42.6(38.6-46.5) | 46.4(42.9-49.8) | 75.9(73.6-78.3) | 88.0(85.8-90.1) | 35.8(32.7-38.9) |
| Mauritania | 49.1(45.3-53.0) | 34.1(30.1-38.0) | 35.5(32.1-38.9) | 67.7(62.9-72.5) | 93.6(92.3-94.9) | 37.7(34.2-41.2) |
| Mozambique | 48.7(42.9-54.4) | 47.2(40.5-53.8) | 40.0(34.0-46.1) | 80.7(75.7-85.7) | 89.7(86.4-93.0) | 34.6(28.2-41.0) |
| Namibia | 39.7(36.0-43.4) | 40.2(37.8-42.6) | 32.2(29.9-34.5) | 57.6(54.6-60.6) | 87.4(85.9-88.9) | 28.6(26.0-31.3) |
| Seychelles | 40.6(37.3-43.9) | 31.5(29.0-34.0) | 40.1(37.5-42.7) | 69.0(66.6-71.4) | 91.9(90.4-93.4) | 22.5(20.4-24.6) |
| Swaziland | 34.3(31.5-37.1) | 41.4(39.4-43.4) | 32.0(30.0-34.1) | 59.0(56.3-61.7) | 82.9(81.3-84.5) | 28.0(25.5-30.6) |
| United Republic of Tanzania | 56.9(53.8-60.1) | 38.0(34.0-42.0) | 38.5(34.6-42.4) | 76.8(74.3-79.2) | 90.4(89.0-91.8) | 35.5(31.9-39.2) |
| **Pooled estimates** | 45.5(39.1-51.9) | 39.0(35.5-42.6) | 37.7(33.7-41.6) | 69.5(63.1-75.9) | 89.1(86.3-92.0) | 31.6(27.1-36.2) |
| **I^2^ (%)** | 95.7 | 89.2 | 91.8 | 97.0 | 95.2 | 93.6 |
| **Eastern Mediterranean Region** | | | | | | |
| Afghanistan | 43.2(35.9-50.4) | 50.9(42.9-58.8) | 52.7(45.1-60.3) | 60.4(55.7-65.0) | 88.1(85.3-90.9) | 66.2(58.0-74.4) |
| Egypt | 43.9(37.8-50.0) | 35.1(29.2-41.0) | 47.8(40.0-55.5) | 75.9(69.7-82.1) | 91.8(89.8-93.7) | 52.0(44.5-59.5) |
| Iraq | 47.6(43.7-51.5) | 41.5(37.5-45.5) | 45.8(41.3-50.3) | 87.5(85.4-89.5) | 94.2(93.1-95.3) | 48.5(44.5-52.5) |
| Kuwait | 35.0(30.4-39.6) | 32.5(29.3-35.7) | 41.7(37.4-46.0) | 69.5(65.7-73.3) | 92.6(90.8-94.4) | 43.0(37.7-48.4) |
| Lebanon | 44.7(40.6-48.9) | 47.7(44.0-51.4) | 53.3(50.1-56.5) | 82.4(79.9-84.8) | 96.6(95.5-97.6) | 70.1(65.9-74.3) |
| Morocco | 45.5(41.9-49.0) | 27.4(24.1-30.6) | 39.2(34.0-44.4) | 74.1(73.0-75.3) | 91.3(89.5-93.1) | 30.5(25.7-35.4) |
| Oman | 49.7(47.2-52.3) | 40.4(37.3-43.4) | 42.8(40.3-45.4) | 85.9(83.7-88.1) | 92.6(91.5-93.6) | 58.7(56.2-61.1) |
| Qatar | 33.3(29.6-37.0) | 25.7(22.8-28.5) | 26.7(23.3-30.1) | 77.3(74.9-79.8) | 87.7(85.5-89.9) | 44.3(39.4-49.1) |
| Syrian Arab Republic | 39.9(36.0-43.7) | 26.4(23.0-29.7) | 37.7(32.9-42.4) | 86.6(84.9-88.3) | 94.9(93.5-96.3) | 44.2(39.1-49.3) |
| United Arab Emirates | 46.5(42.3-50.6) | 46.5(43.4-49.6) | 49.5(46.6-52.4) | 79.1(77.0-81.2) | 93.9(93.0-94.9) | 66.5(62.6-70.4) |
| **Pooled estimates** | 43.0(39.3-46.7) | 37.2(31.5-42.9) | 43.6(38.2-49.0) | 78.1(73.7-82.5) | 92.6(91.2-94.0) | 52.4(44.8-60.2) |
| **I^2^ (%)** | 88.1 | 96.0 | 94.5 | 97.4 | 90.3 | 96.6 |
| **America Region** | | | | | | |
| Antigua and Barbuda | 37.7(33.8-41.6) | 33.0(30.1-35.9) | 40.4(36.8-44.1) | 60.5(57.4-63.6) | 91.3(89.2-93.3) | 19.6(17.2-22.0) |
| Argentina | 30.3(28.9-31.7) | 48.6(46.7-50.5) | 54.4(52.7-56.2) | 67.7(66.3-69.0) | 94.4(93.6-95.2) | 50.8(48.8-52.8) |
| Barbados | 25.7(23.1-28.3) | 29.9(27.4-32.4) | 37.8(35.0-40.6) | 72.3(69.1-75.4) | 94.2(92.9-95.5) | 17.9(16.0-19.9) |
| Belize | 56.4(52.3-60.5) | 48.4(45.3-51.4) | 53.8(50.8-56.7) | 61.0(57.4-64.5) | 92.1(90.5-93.8) | 42.5(40.3-44.6) |
| Bolivia | 40.1(37.7-42.5) | 32.8(30.6-34.9) | 38.0(35.3-40.7) | 78.9(77.5-80.3) | 92.1(91.1-93.1) | 40.5(36.6-44.4) |
| British Virgin Islands | 34.6(32.2-37.0) | 36.6(34.2-39.0) | 46.9(44.5-47.3) | 68.6(66.2-71.0) | 91.5(90.9-92.1) | 22.3(21.6-23.0) |
| Bahamas | 50.2(46.9-53.5) | 36.5(33.4-39.6) | 46.1(42.2-50.1) | 63.6(60.6-66.6) | 92.2(90.3-94.1) | 25.2(21.2-29.2) |
| Costa Rica | 33.8(29.5-38.1) | 46.6(44.8-48.4) | 53.7(50.0-57.4) | 82.0(80.0-83.9) | 94.2(93.1-95.2) | 51.3(47.6-55.1) |
| Curaçao | 33.3(30.2-36.4) | 53.4(50.8-56.0) | 65.3(63.1-67.5) | 78.8(77.0-80.6) | 90.6(89.2-91.9) | 48.1(45.6-50.7) |
| El Salvador | 56.7(51.6-61.7) | 50.9(47.2-54.6) | 57.8(54.3-61.4) | 76.2(73.4-79.0) | 94.5(92.9-96.1) | 54.3(50.9-57.8) |
| Honduras | 52.5(49.2-55.9) | 47.2(43.6-50.9) | 55.7(52.1-59.3) | 81.4(79.1-83.8) | 93.7(92.4-95.0) | 45.5(43.1-48.0) |
| Jamaica | 39.9(35.1-44.6) | 33.7(27.6-39.8) | 39.6(35.1-44.0) | 52.2(46.2-58.2) | 89.6(86.4-92.8) | 22.9(19.0-26.8) |
| Peru | 43.7(40.9-46.5) | 35.2(32.9-37.5) | 36.2(33.3-39.0) | 80.0(77.6-82.4) | 94.7(93.8-95.6) | 43.4(41.3-45.5) |
| Saint Kitts and Nevis | 27.5(26.2-28.8) | 25.8(24.5-27.1) | 32.3(31.0-33.6) | 63.4(61.0-65.8) | 88.8(17.5-90.1) | 14.8(14.2-15.4) |
| Suriname | 36.9(32.4-41.4) | 42.4(39.4-45.3) | 47.4(44.0-50.8) | 68.6(65.6-71.5) | 82.8(80.3-85.3) | 47.1(41.2-53.1) |
| Trinidad and Tobago | 46.0(40.4-51.7) | 39.8(36.0-43.5) | 50.6(46.3-54.9) | 57.1(54.4-59.8) | 91.1(90.2-91.9) | 26.7(22.7-30.8) |
| **Pooled estimates** | 41.6(36.4-46.7) | 41.4(37.1-45.6) | 48.4(43.2-53.6) | 70.2(65.8-74.6) | 92.2(91.0-93.3) | 38.3(31.2-45.4) |
| **I^2^ (%)** | 97.4 | 97.1 | 97.6 | 98.1 | 91.1 | 98.9 |
| **Western Pacific Region** | | | | | | |
| Brunei Darussalam | 14.8(13.0-16.6) | 28.4(26.5-30.3) | 41.0(38.7-43.4) | 67.3(64.2-70.5) | 97.0(96.4-97.7) | 55.7(53.3-58.1) |
| Cook Islands | 31.5(27.0-36.1) | 28.7(25.0-32.4) | 39.7(35.4-44.0) | 62.4(59.2-65.6) | 93.5(91.7-95.3) | 47.6(42.6-52.6) |
| Kiribati | 23.0(20.5-25.5) | 15.2(13.5-16.8) | 27.2(24.6-29.8) | 64.9(60.9-68.9) | 97.7(96.9-98.6) | 26.9(22.8-31.0) |
| Lao People's Democratic Republic | 19.0(15.3-22.7) | 16.7(14.9-18.4) | 31.3(28.5-34.1) | 91.6(90.4-92.8) | 95.2(94.2-96.2) | 19.9(17.5-22.3) |
| Malaysia | 14.2(13.4-15.0) | 31.6(30.1-33.0) | 43.2(42.0-44.5) | 74.1(72.9-75.2) | 96.9(96.6-97.2) | 44.5(42.7-46.2) |
| Mongolia | 46.8(44.9-48.6) | 30.5(28.5-32.5) | 49.1(47.3-50.8) | 77.5(75.7-79.4) | 94.4(93.7-95.1) | 24.8(22.9-26.6) |
| Philippines | 23.9(22.2-25.6) | 28.7(26.7-30.7) | 29.5(27.1-31.8) | 74.4(73.0-75.8) | 95.7(94.3-97.1) | 32.2(29.6-34.9) |
| Samoa | 41.9(38.7-45.0) | 32.4(30.0-34.8) | 30.7(27.5-33.9) | 51.8(46.6-57.0) | 84.4(81.7-87.0) | 34.3(30.2-38.4) |
| Solomon Islands | 37.2(33.1-41.3) | 24.4(20.4-28.4) | 29.5(27.3-31.8) | 40.5(35.7-45.3) | 86.0(82.4-89.5) | 26.6(22.7-30.5) |
| Tokelau | 37.0(36.7-38.3) | 24.7(23.5-25.9) | 29.9(27.7-32.1) | 63.1(61.3-64.9) | 92.4(91.1-93.7) | 31.6(30.2-33.0) |
| Tonga | 39.7(37.2-42.1) | 26.7(24.0-29.4) | 30.6(28.3-32.9) | 56.2(53.0-59.4) | 90.8(89.3-92.2) | 42.1(39.2-44.9) |
| Tuvalu | 28.2(27.1-29.3) | 16.1(15.3-16.9) | 18.3(16.7-19.9) | 88.0(86.0-90.1) | 85.7(84.2-87.3) | 39.2(38.1-40.3) |
| Vanuatu | 28.5(19.8-37.2) | 17.3(11.3-23.4) | 20.4(17.2-23.6) | 56.3(49.2-63.5) | 85.1(79.6-90.6) | 28.7(22.4-35.0) |
| Vietnam | 27.7(24.9-30.5) | 30.4(27.6-33.2) | 37.5(34.1-40.8) | 63.2(61.3-65.1) | 94.4(93.2-95.6) | 50.3(46.4-54.2) |
| Wallis and Futuna | 50.1(46.4-53.8) | 37.5(34.3-40.7) | 45.7(41.6-49.8) | 78.3(75.2-81.4) | 96.4(95.1-97.7) | 61.9(57.3-66.5) |
| **Pooled estimates** | 30.6(23.1-38.1) | 26.9(23.0-30.8) | 35.0(30.3-39.8) | 66.2(59.7-72.8) | 93.7(92.4-95.0) | 38.1(31.0-45.3) |
| **I^2^ (%)** | 99.3 | 97.4 | 98.0 | 99.2 | 95.4 | 98.7 |
| **South-East Asia Region** | | | | | | |
| Bangladesh | 54.8(49.4-60.3) | 47.8(42.5-53.1) | 43.9(39.7-48.2) | 85.4(82.3-88.4) | 91.7(89.5-93.8) | 56.7(52.3-61.2) |
| Indonesia | 35.8(32.7-38.9) | 34.8(33.4-36.2) | 39.9(37.6-42.3) | 56.8(55.1-58.6) | 97.1(96.5-97.7) | 39.1(36.9-41.4) |
| Maldives | 27.8(25.4-30.2) | 32.6(30.9-34.4) | 46.7(44.5-49.0) | 66.6(64.4-68.7) | 91.1(89.9-92.3) | 59.7(57.5-61.8) |
| Thailand | 27.9(24.9-30.9) | 28.7(25.9-31.5) | 43.4(40.2-46.5) | 67.6(65.9-69.3) | 93.9(93.0-94.8) | 39.7(36.0-43.4) |
| Timor-Leste | 29.1(26.7-31.6) | 11.5(9.7-13.3) | 23.6(20.8-26.3) | 71.9(68.7-75.1) | 95.6(94.6-96.5) | 27.7(24.8-30.6) |
| **Pooled estimates** | 34.7(28.1-41.3) | 30.9(20.7-41.2) | 39.5(31.1-47.8) | 69.6(61.4-77.8) | 94.0(91.7-96.2) | 44.6(32.2-57.0) |
| **I^2^ (%)** | 95.8 | 99.2 | 97.8 | 98.6 | 96.1 | 98.9 |
| **Total estimates** | 39.6(34.9-44.3) | 35.4(29.3-41.4) | 40.8(35.9-45.7) | 71.4(66.2-76.6) | 92.6(91.4-93.8) | 40.5(32.9-48.1) |
| **I^2^ (%)** | 71.2 | 87.1 | 76.8 | 64.6 | 61.6 | 81.9 |

**Supplementary Table 3**

The prevalence of individual parental and peer support among in-school male adolescents by region, and country groups (Low-income and middle-income countries, 2009–2015).

|  | **Parental support** | | | | **Peer support** | |
| --- | --- | --- | --- | --- | --- | --- |
|  | **Parents checked homework** | **Parents understood problems** | **Parents concerned free time** | **Parents respected for privacy** | **Having close friendships** | **Having supportive classmates** |
| **Africa Region** | | | | | | |
| Benin | 47.2(43.5-50.9) | 41.5(36.4-46.6) | 43.7(40.1-47.2) | 76.2(73.7-78.7) | 90.3(87.7-93.0) | 37.0(33.9-40.2) |
| Mauritania | 49.2(44.5-53.8) | 34.1(29.3-38.9) | 33.9(29.1-38.6) | 66.1(59.2-73.1) | 94.0(91.9-96.1) | 37.1(31.5-42.7) |
| Mozambique | 44.9(40.1-49.7) | 46.8(39.2-54.3) | 37.5(30.2-44.9) | 81.6(75.9-87.3) | 91.4(88.9-94.0) | 35.0(27.3-42.7) |
| Namibia | 40.0(36.5-43.6) | 38.9(36.1-41.8) | 31.4(29.0-33.8) | 58.3(55.2-61.5) | 87.8(85.9-89.7) | 27.7(25.0-30.5) |
| Seychelles | 39.0(35.2-42.9) | 30.7(27.3-34.1) | 37.1(33.6-40.7) | 69.3(66.3-72.4) | 92.3(90.1-94.4) | 21.4(18.4-24.4) |
| Swaziland | 32.2(29.1-35.3) | 38.3(35.8-40.7) | 28.4(26.6-30.3) | 60.3(57.3-63.2) | 82.5(80.7-84.4) | 26.4(22.8-29.9) |
| United Republic of Tanzania | 54.8(51.1-58.6) | 37.0(32.7-41.4) | 36.1(31.8-40.5) | 77.5(75.1-79.8) | 90.0(88.2-91.8) | 35.3(31.1-39.5) |
| **Pooled estimates** | 43.8(37.8-49.9) | 37.6(34.5-40.7) | 35.3(31.0-39.6) | 69.9(63.3-76.5) | 89.7(86.7-92.7) | 31.2(26.2-36.1) |
| **I^2^ (%)** | 94.2 | 77.6 | 91.3 | 96.6 | 92.9 | 91.5 |
| **Eastern Mediterranean Region** | | | | | | |
| Afghanistan | 39.4(29.5-49.3) | 45.5(33.8-57.2) | 47.6(38.9-56.3) | 60.5(53.7-67.3) | 89.5(85.5-93.6) | 62.1(49.7-74.6) |
| Egypt | 41.8(35.1-48.5) | 34.8(26.9-42.7) | 41.3(32.8-49.8) | 70.9(62.2-79.5) | 91.8(88.9-94.7) | 47.8(37.9-57.7) |
| Iraq | 49.7(44.4-55.0) | 42.9(37.5-48.4) | 45.7(39.5-51.8) | 86.4(84.1-88.7) | 94.7(92.9-96.6) | 45.1(40.2-50.0) |
| Kuwait | 41.1(36.3-45.9) | 33.3(29.9-36.7) | 41.5(35.8-47.2) | 69.9(65.4-74.5) | 92.3(89.7-94.8) | 41.0(35.1-47.0) |
| Lebanon | 48.2(43.3-53.1) | 45.7(41.2-50.2) | 49.4(45.2-53.6) | 79.1(75.1-83.2) | 96.5(94.8-98.2) | 66.7(60.5-72.8) |
| Morocco | 43.2(40.1-46.3) | 24.8(21.6-27.9) | 37.3(31.1-43.4) | 72.5(70.2-74.7) | 92.8(91.0-94.5) | 28.1(23.9-32.2) |
| Oman | 47.3(44.1-50.6) | 35.8(32.5-39.1) | 37.7(35.0-40.3) | 82.5(79.8-85.2) | 92.0(90.8-93.3) | 53.0(50.3-55.7) |
| Qatar | 34.8(30.5-39.1) | 23.4(20.2-26.7) | 24.2(20.0-28.3) | 72.4(69.3-75.5) | 83.4(79.1-87.7) | 32.6(28.8-36.5) |
| Syrian Arab Republic | 38.4(34.4-42.3) | 24.8(21.6-27.9) | 34.3(29.0-39.6) | 84.9(82.8-86.9) | 95.5(94.0-97.0) | 40.5(33.8-47.3) |
| United Arab Emirates | 53.4(49.3-57.5) | 45.4(42.1-48.7) | 46.7(43.8-49.5) | 77.4(75.1-79.8) | 93.0(91.7-94.2) | 58.2(54.2-62.2) |
| **Pooled estimates** | 43.9(40.2-47.6) | 35.3(29.5-41.1) | 40.4(35.3-45.6) | 76.1(71.9-80.4) | 92.7(91.2-94.2) | 47.2(39.3-55.1) |
| **I^2^ (%)** | 84.9 | 95.3 | 91.9 | 94.9 | 83.4 | 96.2 |
| **America Region** | | | | | | |
| Antigua and Barbuda | 38.6(33.4-43.8) | 31.6(27.5-35.7) | 36.7(31.8-41.5) | 57.8(51.9-63.7) | 88.7(85.7-91.8) | 18.4(15.1-21.7) |
| Argentina | 32.6(30.4-34.9) | 47.4(45.0-49.7) | 50.2(48.0-52.3) | 69.5(67.8-71.2) | 93.2(91.7-94.6) | 47.3(44.9-49.7) |
| Barbados | 27.9(24.3-31.5) | 31.1(27.8-34.4) | 35.4(31.8-39.0) | 69.9(65.6-74.3) | 92.1(90.1-94.1) | 17.8(15.0-20.7) |
| Belize | 57.8(53.7-62.0) | 50.7(47.3-54.2) | 54.2(50.5-57.9) | 62.5(57.9-67.1) | 92.2(90.0-94.3) | 39.3(35.7-43.0) |
| Bolivia | 39.0(36.1-42.0) | 31.9(29.0-34.9) | 36.1(32.9-39.3) | 79.3(77.7-80.9) | 92.1(90.7-93.6) | 38.7(33.8-43.7) |
| British Virgin Islands | 35.8 (33.5-38.1) | 39.5(37.2-41.8) | 41.5(39.2-43.7) | 67.3(65.1-69.5) | 89.7(87.1-92.3) | 21.3(19.2-23.4) |
| Bahamas | 48.5(44.2-52.9) | 36.8(31.3-42.2) | 44.3(38.3-50.4) | 64.9(62.6-67.2) | 91.7(89.2-94.2) | 23.7(18.8-28.5) |
| Costa Rica | 35.5(30.5-40.5) | 44.8(41.7-48.0) | 50.7(46.7-54.8) | 82.8(80.3-85.3) | 94.0(92.2-95.8) | 47.5(43.3-51.8) |
| Curaçao | 36.5(32.5-40.5) | 52.0(47.8-56.2) | 62.9(60.0-65.8) | 77.5(74.5-80.5) | 90.6(88.6-92.5) | 47.0(43.8-50.2) |
| El Salvador | 55.5(50.2-60.8) | 53.2(49.3-57.0) | 56.8(52.3-61.2) | 76.0(72.7-79.3) | 93.1(90.8-95.3) | 51.8(47.2-56.5) |
| Honduras | 53.2(49.5-56.9) | 49.2(45.3-53.1) | 55.8(51.3-60.2) | 83.1(80.5-85.7) | 94.2(92.2-96.2) | 40.8(36.7-44.9) |
| Jamaica | 39.4(34.5-44.2) | 35.4(28.6-42.2) | 36.6(32.9-40.4) | 53.4(48.2-58.6) | 89.1(84.3-94.0) | 23.2(19.1-27.3) |
| Peru | 42.2(38.1-46.2) | 33.6(29.7-37.4) | 32.8(29.4-36.3) | 81.1(77.7-84.5) | 94.6(93.3-95.9) | 36.8(33.7-40.0) |
| Saint Kitts and Nevis | 30.0(27.3-32.7) | 25.0(23.1-27.0) | 28.9(26.2-31.6) | 63.2(61.1-65.3) | 87.1(84.8-89.4) | 15.2(13.1-17.3) |
| Suriname | 39.3(35.7-42.8) | 43.2(38.5-48.0) | 43.6(38.7-48.4) | 69.6(65.6-73.5) | 84.3(80.7-87.9) | 43.4(36.6-50.2) |
| Trinidad and Tobago | 48.1(42.6-53.6) | 39.6(35.4-43.7) | 48.6(42.9-54.4) | 56.7(53.7-59.7) | 91.5(90.7-92.3) | 25.8(23.0-28.6) |
| **Pooled estimates** | 42.4(37.7-47.1) | 41.5(37.1-45.9) | 46.1(40.7-51.4) | 70.5(65.8-75.2) | 92.0(91.0-93.0) | 35.8(29.3-42.2) |
| **I^2^ (%)** | 95.1 | 94.9 | 96.5 | 97.1 | 75.9 | 97.8 |
| **Western Pacific Region** | | | | | | |
| Brunei Darussalam | 14.9(12.5-17.3) | 31.3(27.9-34.6) | 41.9(38.2-45.6) | 66.8(62.8-70.8) | 96.6(95.3-97.8) | 52.2(48.6-55.7) |
| Cook Islands | 33.3(27.7-38.9) | 30.5(25.1-35.9) | 41.6(35.4-47.8) | 63.6(59.2-68.0) | 93.1(90.2-96.0) | 49.9(42.2-57.6) |
| Kiribati | 20.0(16.3-23.8) | 15.1(11.8-18.5) | 23.7(19.7-27.6) | 63.7(57.7-69.7) | 97.5(96.3-98.7) | 23.2(17.7-28.7) |
| Lao People's Democratic Republic | 20.4(16.2-24.7) | 16.7(14.2-19.1) | 30.8(26.7-34.9) | 92.0(90.2-93.8) | 94.0(92.5-95.5) | 19.4(16.5-22.3) |
| Malaysia | 15.4(14.4-16.4) | 32.1(30.2-33.9) | 41.6(39.8-43.4) | 71.7(70.2-73.2) | 96.6(96.1-97.1) | 36.5(34.4-38.5) |
| Mongolia | 46.7(44.4-48.9) | 29.2(26.8-31.6) | 49.3(47.1-51.5) | 77.6(75.5-79.8) | 95.1(94.1-96.1) | 25.8(23.5-28.1) |
| Philippines | 25.7(23.6-27.9) | 29.0(27.2-30.8) | 27.5(24.7-30.3) | 74.1(72.5-75.8) | 95.4(94.3-96.4) | 32.2(29.8-34.6) |
| Samoa | 33.0(28.1-37.8) | 28.6(24.8-32.4) | 24.2(20.8-27.7) | 56.1(50.6-61.5) | 83.9(80.9-86.9) | 32.0(27.1-36.9) |
| Solomon Islands | 34.9(30.0-39.9) | 21.2(16.4-26.0) | 28.6(25.7-31.5) | 40.0(33.6-46.3) | 84.0(78.1-89.9) | 26.5(22.8-30.1) |
| Tokelau | 42.7(39.2-46.6) | 26.2(24.1-28.3) | 30.5(27.6-33.4) | 58.1(55.9-60.3) | 89.3(86.5-92.1) | 31.5(28.6-34.4) |
| Tonga | 40.6(37.4-43.9) | 24.9(21.3-28.5) | 31.7(28.0-35.4) | 59.2(54.2-64.1) | 90.2(88.1-92.3) | 42.4(37.8-47.1) |
| Tuvalu | 25.7(23.2-28.2) | 14.3(12.0-16.3) | 16.3(14.6-18.6) | 84.6(82.1-87.1) | 85.9(83.4-88.4) | 34.8(32.4-37.2) |
| Vanuatu | 23.6(17.2-30.0) | 17.1(8.8-25.4) | 18.6(13.5-23.6) | 56.1(49.9-62.3) | 84.8(78.8-90.8) | 27.5(21.2-33.8) |
| Vietnam | 31.3(28.0-34.6) | 32.7(30.2-35.1) | 38.2(34.7-41.6) | 60.1(57.5-62.7) | 93.7(92.0-95.5) | 50.2(45.6-54.9) |
| Wallis and Futuna | 54.5(49.0-59.9) | 38.5(33.7-43.4) | 44.8(39.7-49.9) | 77.5(73.0-81.9) | 95.3(92.9-97.7) | 59.6(53.7-65.5) |
| **Pooled estimates** | 30.3(23.1-37.5) | 26.8(23.3-30.4) | 34.0(28.8-39.3) | 66.3(59.9-72.6) | 93.3(91.9-94.8) | 36.6(30.6-42.5) |
| **I^2^ (%)** | 98.8 | 94.4 | 97.0 | 98.5 | 92.1 | 97.3 |
| **South-East Asia Region** | | | | | | |
| Bangladesh | 54.0(49.1-59.0) | 43.9(36.9-50.9) | 40.8(34.8-46.9) | 83.1(79.6-86.6) | 93.4(90.8-96.1) | 56.1(50.7-61.6) |
| Indonesia | 36.3(33.1-39.5) | 31.6(29.8-33.4) | 31.1(28.8-33.3) | 56.9(54.8-59.1) | 96.4(95.6-97.3) | 34.0(31.3-36.7) |
| Maldives | 30.7(27.5-33.8) | 32.2(29.3-35.1) | 44.5(41.1-47.9) | 66.2(62.6-69.9) | 90.4(88.4-92.5) | 56.8(53.0-60.5) |
| Thailand | 27.7(24.3-31.1) | 24.8(21.0-28.6) | 36.6(33.4-39.8) | 68.5(64.7-72.3) | 92.4(90.7-94.1) | 31.8(28.2-35.4) |
| Timor-Leste | 28.3(25.4-31.2) | 11.2(9.5-13.0) | 22.7(19.6-25.8) | 70.8(67.0-74.6) | 95.9(94.6-97.2) | 25.6(22.5-28.7) |
| **Pooled estimates** | 35.2(27.8-42.6) | 28.5(17.7-39.3) | 35.0(27.7-42.3) | 69.1(59.6-78.5) | 93.8(91.6-96.0) | 40.8(29.1-52.4) |
| **I^2^ (%)** | 95.8 | 98.8 | 96.0 | 97.6 | 90.5 | 98.2 |
| **Total estimates** | 39.8(35.1-44.4) | 34.3(28.4-40.3) | 38.2(33.7-42.7) | 71.0(67.3-74.6) | 92.5(91.6-93.5) | 37.6(32.2-42.9) |
| **I^2^ (%)** | 72.1 | 87.8 | 71.5 | 48.2 | 41.8 | 66.7 |

**Supplementary Table 4**

The prevalence of individual parental and peer support among in-school female adolescents by region, and country groups (Low-income and middle-income countries, 2009–2015).

|  | **Parental support** | | | | **Peer support** | |
| --- | --- | --- | --- | --- | --- | --- |
|  | **Parents checked homework** | **Parents understood problems** | **Parents concerned free time** | **Parents respected for privacy** | **Having close friendships** | **Having supportive classmates** |
| **Africa Region** | | | | | |  |
| Benin | 54.1(50.0-58.2) | 44.5(41.3-47.8) | 51.4(45.5-57.2) | 75.2(71.4-79.1) | 83.2(80.1-86.3) | 32.9(28.9-36.9) |
| Mauritania | 48.5(43.9-53.0) | 33.2(29.2-37.3) | 37.0(32.6-41.4) | 69.7(65.2-74.1) | 93.1(91.3-95.0) | 38.5(33.8-43.1) |
| Mozambique | 51.6(43.7-59.6) | 46.1(39.4-52.7) | 41.7(34.6-48.7) | 79.7(73.9-85.5) | 86.4(80.2-92.6) | 33.7(26.4-41.0) |
| Namibia | 39.2(34.7-43.6) | 41.2(38.3-44.1) | 33.2(30.2-36.2) | 57.1(53.8-60.5) | 86.9(85.2-88.7) | 29.3(26.2-32.5) |
| Seychelles | 42.0(37.9-46.1) | 32.2(29.1-35.4) | 42.9(39.6-46.3) | 68.7(65.6-71.9) | 91.6(89.8-93.3) | 23.5(20.6-26.4) |
| Swaziland | 36.5(32.7-40.2) | 44.3(41.4-47.3) | 35.7(32.5-38.8) | 57.6(54.6-60.6) | 83.0(80.5-85.5) | 29.6(26.7-32.4) |
| United Republic of Tanzania | 58.6(55.0-62.2) | 38.5(34.0-42.9) | 40.5(35.5-45.4) | 76.0(72.5-79.4) | 90.4(88.6-92.2) | 35.4(31.3-39.5) |
| **Pooled estimates** | 47.1(40.4-53.9) | 39.9(35.7-44.0) | 40.0(35.8-44.2) | 69.0(62.4-75.6) | 88.0(85.1-90.9) | 31.6(27.8-35.3) |
| **I^2^ (%)** | 94.0 | 88.9 | 85.8 | 95.6 | 91.6 | 85.7 |
| **Eastern Mediterranean Region** | | | | | | |
| Afghanistan | 47.1(41.9-52.2) | 56.3(49.4-63.1) | 57.7(51.4-63.9) | 61.1(55.1-67.0) | 85.8(81.8-89.7) | 70.1(63.5-76.6) |
| Egypt | 45.5(38.1-52.9) | 34.9(28.7-41.2) | 54.1(45.0-63.2) | 80.8(73.4-88.3) | 91.6(89.4-93.9) | 56.1(48.8-63.3) |
| Iraq | 44.6(39.8-49.5) | 39.2(35.2-43.1) | 46.0(40.2-51.8) | 88.9(86.1-91.7) | 93.4(92.1-94.7) | 52.8(47.0-58.6) |
| Kuwait | 28.6(25.4-31.7) | 31.6(26.7-36.5) | 41.3(36.3-46.3) | 69.0(65.0-73.1) | 92.5(89.9-95.1) | 44.5(37.0-52.1) |
| Lebanon | 41.8(37.2-46.4) | 49.4(44.9-53.8) | 56.6(52.7-60.6) | 85.1(82.5-87.7) | 96.6(95.6-97.7) | 73.1(68.4-77.8) |
| Morocco | 48.3(42.9-53.8) | 30.8(26.1-35.5) | 41.5(36.9-46.1) | 76.2(74.0-78.3) | 89.4(86.7-92.1) | 33.5(27.0-39.9) |
| Oman | 52.1(49.0-55.1) | 44.6(40.8-48.5) | 47.6(44.7-50.5) | 88.7(86.4-91.0) | 92.8(91.3-94.3) | 63.2(60.1-66.4) |
| Qatar | 32.0(26.8-37.2) | 27.4(23.0-31.7) | 28.5(23.6-33.5) | 81.3(77.9-84.8) | 90.9(88.7-93.0) | 53.0(46.1-59.9) |
| Syrian Arab Republic | 41.4(37.6-45.2) | 28.0(24.0-31.9) | 41.1(36.0-46.2) | 88.4(86.3-90.5) | 94.3(92.3-96.3) | 47.9(41.3-54.6) |
| United Arab Emirates | 41.8(37.6-46.0) | 47.1(43.1-51.1) | 51.4(47.6-55.2) | 79.9(76.4-83.3) | 94.4(93.3-95.5) | 72.3(68.6-76.1) |
| **Pooled estimates** | 42.3(36.9-47.6) | 38.8(33.0-44.6) | 46.4(41.3-51.6) | 80.2(75.6-84.8) | 92.5(91.0-94.1) | 56.8(49.1-64.6) |
| **I^2^ (%)** | 93.4 | 93.9 | 91.9 | 95.7 | 86.8 | 95.2 |
| **America Region** | | | | | | |
| Antigua and Barbuda | 37.1(32.6-41.6) | 33.5(28.7-38.4) | 44.7(39.6-49.8) | 62.7(58.6-66.8) | 94.2(91.8-96.5) | 21.5(18.0-25.1) |
| Argentina | 28.1(26.4-29.8) | 49.8(47.6-51.9) | 58.2(56.0-60.4) | 66.1(63.9-68.3) | 95.3(94.5-96.1) | 53.9(51.7-56.2) |
| Barbados | 23.6(20.2-26.9) | 28.7(25.2-32.2) | 40.1(36.5-43.8) | 74.5(70.3-78.7) | 96.1(94.7-97.5) | 18.1(15.7-20.5) |
| Belize | 55.1(49.9-60.3) | 46.1(42.3-49.9) | 53.2(49.6-56.8) | 59.6(55.8-63.4) | 92.0(89.8-94.3) | 45.1(42.3-48.0) |
| Bolivia | 41.5(38.2-44.8) | 33.8(31.0-36.7) | 39.8(36.8-42.7) | 78.7(76.6-80.7) | 92.4(91.1-93.7) | 42.3(38.2-46.4) |
| British Virgin Islands | 33.3(31.1-35.5) | 33.7(31.5-35.9) | 51.6(50.1-53.1) | 69.7(67.7-71.8) | 93.0(91.3-94.7) | 23.0(21.2-24.8) |
| Bahamas | 51.7(47.4-56.0) | 36.1(32.0-40.3) | 47.7(43.1-52.2) | 62.5(57.6-67.3) | 92.6(89.8-95.4) | 26.4(21.7-31.2) |
| Costa Rica | 32.1(27.8-36.3) | 48.2(45.3-51.1) | 56.5(52.5-60.5) | 81.2(78.8-83.5) | 94.4(92.4-96.3) | 55.2(50.6-59.7) |
| Curaçao | 30.2(27.1-33.4) | 54.3(51.3-57.3) | 67.5(64.8-70.2) | 80.0(77.9-82.1) | 90.7(89.1-92.3) | 49.1(45.8-52.3) |
| El Salvador | 58.0(52.1-63.9) | 48.4(43.2-53.7) | 58.7(54.1-63.3) | 76.6(72.5-80.7) | 96.0(94.7-97.3) | 56.5(52.7-60.3) |
| Honduras | 51.8(47.1-56.6) | 45.7(41.3-50.1) | 55.7(51.5-59.8) | 79.8(76.1-83.6) | 93.1(91.3-94.9) | 49.4(46.0-52.9) |
| Jamaica | 40.2(32.2-48.1) | 32.0(22.1-41.8) | 42.5(36.2-48.7) | 51.0(43.0-58.9) | 89.7(85.7-93.6) | 22.4(18.4-26.4) |
| Peru | 45.2(41.7-48.6) | 36.6(34.5-38.7) | 39.2(35.5-42.9) | 78.9(76.2-81.6) | 94.9(93.8-95.9) | 50.1(47.5-52.6) |
| Saint Kitts and Nevis | 25.2(23.1-27.3) | 26.5(24.5-28.5) | 35.6(33.1-38.1) | 63.5(61.2-65.7) | 90.4(89.0-91.8) | 14.4(12.0-16.8) |
| Suriname | 34.8(28.5-41.1) | 41.9(38.4-45.3) | 51.2(46.2-56.2) | 67.7(64.2-71.2) | 81.5(78.8-84.1) | 50.7(43.3-58.0) |
| Trinidad and Tobago | 44.0(35.5-52.6) | 39.8(34.6-44.9) | 52.4(47.9-56.9) | 57.4(53.6-61.2) | 90.4(89.0-91.8) | 27.6(22.5-32.7) |
| **Pooled estimates** | 40.8(35.1-46.6) | 41.2(37.0-45.5) | 50.6(45.5-55.7) | 70.1(65.5-74.6) | 92.6(91.0-94.1) | 40.6(32.8-48.4) |
| **I^2^ (%)** | 96.7 | 95.4 | 96.2 | 96.6 | 92.4 | 98.6 |
| **Western Pacific Region** | | | | | | |
| Brunei Darussalam | 14.8(12.4-17.2) | 25.6(23.2-28.0) | 40.3(37.7-43.0) | 67.8(64.2-71.5) | 97.5(96.7-98.4) | 59.1(55.7-62.5) |
| Cook Islands | 30.1(24.8-35.4) | 26.6(22.2-31.1) | 38.0(32.7-43.2) | 61.1(56.2-66.0) | 94.0(91.9-96.2) | 45.9(40.1-51.7) |
| Kiribati | 25.6(22.2-29.0) | 15.1(12.6-17.5) | 30.2(27.6-32.8) | 66.2(62.9-69.4) | 98.0(97.1-98.9) | 30.3(25.9-34.7) |
| Lao People's Democratic Republic | 17.3(13.9-20.8) | 16.4(14.3-18.5) | 31.9(29.1-34.7) | 91.2(90.0-92.3) | 96.7(95.4-97.9) | 20.5(17.2-23.8) |
| Malaysia | 13.0(12.1-13.9) | 31.1(29.5-32.7) | 44.9(43.4-46.4) | 76.4(75.1-77.7) | 97.2(96.8-97.6) | 52.5(50.6-54.4) |
| Mongolia | 46.9(43.9-49.8) | 31.8(29.2-34.5) | 48.8(46.4-51.1) | 48.8(46.4-51.1) | 93.7(92.7-94.7) | 23.9(21.4-26.3) |
| Philippines | 22.1(20.0-24.2) | 28.4(25.5-31.2) | 31.3(28.5-34.2) | 74.6(72.7-76.6) | 96.0(94.0-97.9) | 32.2(28.4-36.0) |
| Samoa | 48.7(44.9-52.6) | 34.9(32.6-37.2) | 35.6(30.7-40.4) | 47.6(41.6-53.7) | 85.2(81.7-88.6) | 36.0(31.2-40.9) |
| Solomon Islands | 38.9(33.1-44.6) | 28.6(22.9-34.4) | 30.4(25.9-35.0) | 42.8(38.0-47.5) | 87.4(84.0-90.9) | 27.1(18.9-35.4) |
| Tokelau | 32.0(30.1-33.9) | 23.3(21.0-25.6) | 27.9(26.5-29.3) | 66.3(65.0-67.6) | 94.8(92.4-97.2) | 32.7(30.2-35.2) |
| Tonga | 38.2(34.8-41.7) | 28.6(25.4-31.9) | 29.5(26.5-32.6) | 53.3(49.5-57.1) | 91.4(89.3-93.4) | 41.6(38.1-45.0) |
| Tuvalu | 30.3(28.3-32.3) | 17.6(15.3-19.9) | 20.2(18.2-22.2) | 90.9(88.6-93.2) | 85.4(83.4-87.4) | 42.5(40.5-44.5) |
| Vanuatu | 34.3(20.8-47.9) | 17.1(11.5-22.7) | 22.3(17.4-27.1) | 56.5(45.8-67.3) | 85.2(79.1-91.2) | 29.9(21.7-38.1) |
| Vietnam | 24.5(21.3-27.7) | 28.5(24.7-32.2) | 36.8(32.6-41.0) | 65.9(63.3-68.5) | 95.0(93.8-96.2) | 50.4(46.0-54.8) |
| Wallis and Futuna | 46.7(42.0-51.4) | 36.7(32.3-41.0) | 46.3(41.4-51.3) | 78.6(74.6-82.7) | 97.3(95.9-98.8) | 63.7(58.2-69.3) |
| **Pooled estimates** | 30.7(23.3-38.2) | 26.9(22.9-30.9) | 35.9(31.5-40.3) | 64.1(55.6-72.5) | 94.4(93.0-95.7) | 39.5(31.4-47.6) |
| **I^2^ (%)** | 98.9 | 96.1 | 96.4 | 99.3 | 93.2 | 98.4 |
| **South-East Asia Region** | | | | | | |
| Bangladesh | 56.5(50.3-62.6) | 55.0(49.1-60.8) | 49.8(44.3-55.2) | 89.6(86.4-92.8) | 88.5(85.9-91.1) | 57.9(51.2-64.7) |
| Indonesia | 35.3(31.6-39.0) | 37.8(36.2-39.4) | 48.3(45.6-50.9) | 56.7(54.5-59.0) | 97.7(97.2-98.3) | 44.0(41.6-46.3) |
| Maldives | 25.0(22.3-27.7) | 32.7(30.0-35.3) | 48.3(44.9-51.7) | 67.1(64.8-69.3) | 91.6(90.4-92.7) | 61.9(59.4-64.4) |
| Thailand | 28.1(24.9-31.2) | 31.9(29.1-34.6) | 48.9(45.3-52.5) | 66.7(64.3-69.2) | 95.1(94.0-96.1) | 46.0(41.5-50.5) |
| Timor-Leste | 29.8(27.1-32.6) | 12.0(9.4-14.6) | 24.1(20.6-27.6) | 72.8(69.5-76.2) | 94.8(93.5-96.1) | 29.4(26.0-32.9) |
| **Pooled estimates** | 34.5(27.2-41.8) | 33.7(22.9-44.4) | 43.8(34.1-53.6) | 70.5(60.7-80.4) | 93.7(90.9-96.5) | 47.8(35.9-59.6) |
| **I^2^ (%)** | 95.7 | 98.8 | 97.4 | 98.6 | 96.9 | 98.4 |
| **Total estimates** | 38.6(32.7-44.5) | 36.2(30.0-42.5) | 41.6(36.1-47.1) | 70.1(63.8-76.3) | 92.7(91.1-94.3) | 41.4(32.5-50.2) |
| **I^2^ (%)** | 76.2 | 91.2 | 85.9 | 80.4 | 79.7 | 88.4 |

**Supplementary Table 5**

The prevalence of individual parental and peer support among in-school low socioeconomic status adolescents by region, and country groups (Low-income and middle-income countries, 2009–2015).

|  | **Parental support** | | | | **Peer support** | |
| --- | --- | --- | --- | --- | --- | --- |
|  | **Parents checked homework** | **Parents understood problems** | **Parents concerned free time** | **Parents respected for privacy** | **Having close friendships** | **Having supportive classmates** |
| **Africa Region** | | | | | |  |
| Benin | 52.0(47.7-56.2) | 43.7(37.2-50.2) | 49.0(43.2-54.7) | 74.3(70-78.6) | 85.0(82.1-87.9) | 40.1(34.8-45.5) |
| Mauritania | 42.7(34.2-51.2) | 31.7(24.2-39.3) | 32.2(21.7-42.6) | 56.6(46.9-66.3) | 92.6(88.7-96.6) | 35.4(28.0-42.8) |
| Mozambique | 56.8(49.2-64.4) | 46.1(34.1-58.1) | 43.9(31.7-56.1) | 81.5(74.4-88.7) | 92.3(90.3-94.4) | 35.8(28.3-43.2) |
| Namibia | 45.1(37.7-52.4) | 38.3(32.4-44.3) | 29.2(24.6-33.9) | 52.0(46.1-57.9) | 83.6(78.7-88.6) | 29.4(25.4-33.5) |
| Seychelles | 28.9(22.6-35.1) | 25.6(19.1-32.2) | 29.3(22.6-36.0) | 68.3(62.0-74.5) | 89.8(85.8-93.8) | 16.4(11.5-21.2) |
| Swaziland | 37(30.3-43.6) | 33.8(27.8-39.9) | 36.1(31.3-41.0) | 53.9(46.4-61.4) | 77.8(72.8-82.8) | 24.6(18.2-31.1) |
| United Republic of Tanzania | 56.4(48.3-64.4) | 32.1(25.2-39) | 39.2(27.8-50.5) | 71.4(63.9-79) | 87.9(83.6-92.3) | 32.7(24-41.4) |
| **Eastern Mediterranean Region** | | | | | | |
| Afghanistan | 39.6(30.1-49.1) | 44.1(27.8-60.5) | 46.9(32.2-61.6) | 50.0(43.6-56.3) | 89.2(84.3-94.1) | 70.9(65.6-76.3) |
| Egypt | 27.6(21.6-33.5) | 30.7(22.8-38.6) | 36.6(28.5-44.7) | 76.0(69.7-82.3) | 82.1(73.8-90.3) | 33.5(25.8-41.2) |
| Iraq | 37.8(32.4-43.2) | 26.4(21.6-31.1) | 35.6(27.8-43.3) | 83.7(78.0-89.3) | 90.5(84.8-96.2) | 38.1(30.7-45.5) |
| Kuwait | 29.9(20.1-39.8) | 34.7(26.7-42.7) | 31(24.1-37.9) | 59.5(49.6-69.4) | 90.9(88.0-93.8) | 36.4(27.5-45.3) |
| Lebanon | 42.3(35.0-49.5) | 31.6(22.3-40.9) | 40.7(35.3-46.1) | 68.4(58.7-78.1) | 87.4(80.3-94.5) | 53.5(45.1-61.8) |
| Morocco | 35.0(25.8-44.2) | 23.1(16.3-30.0) | 33.9(24.9-42.9) | 70.5(63.7-77.3) | 89.0(84.3-93.7) | 21.5(17.0-26.0) |
| Oman | 36.6(29.4-43.8) | 26.9(19.8-33.9) | 34.1(27.0-41.2) | 74.2(67.5-81.0) | 90.8(85.3-96.3) | 46.8(37.9-55.8) |
| Qatar | 29.6(22.3-36.9) | 15.8(7.0-24.6) | 24.0(16.9-31.1) | 64.8(55.3-74.3) | 84.9(76.9-93.0) | 42.0(32.8-51.2) |
| Syrian Arab Republic | 31.1(25.8-36.4) | 20.1(14.8-25.4) | 29.5(23.4-35.5) | 79.0(72.6-85.3) | 93.4(90.5-96.4) | 31.3(24.1-38.5) |
| United Arab Emirates | 48.5(36.1-61.0) | 27.8(19.4-36.1) | 32.6(25.7-39.5) | 79.2(73.3-85.1) | 92.1(86.8-97.4) | 50.1(38.8-61.4) |
| **America Region** | | | | | | |
| Antigua and Barbuda | 44.4(32.7-56.0) | 36.4(25.8-47.0) | 40.4(30.8-50.0) | 59.1(50.6-67.5) | 86.8(80.1-93.5) | 14.8(9.4-20.3) |
| Argentina | 24.0(20.0-28.1) | 32.9(27.7-38.1) | 33.4(29.2-37.5) | 56(48.2-63.9) | 90.8(88.7-93.0) | 36.9(32.4-41.4) |
| Barbados | 23.8(12.7-34.9) | 24.6(16.6-32.6) | 37.5(26.2-48.9) | 73.1(60.0-86.1) | 89.7(82.6-96.8) | 24.1(14.6-33.5) |
| Belize | 54.8(44.5-65.0) | 38.6(28.1-49.1) | 46.3(37.9-54.7) | 56.4(43.4-69.5) | 89(83.1-94.9) | 31.9(25.0-38.8) |
| Bolivia | 37.2(31.4-43.1) | 31.8(28.1-35.6) | 32.2(27.9-36.6) | 78.5(73.5-83.6) | 88.9(85.6-92.3) | 35.7(29.9-41.4) |
| British Virgin Islands | 21.9 | 20.1 | 29.5 | 62.4 | 91.8 | 17.3 |
| Bahamas | 46.5(37.2-55.9) | 20.7(15.3-26.2) | 41.1(33.5-48.7) | 50(40.6-59.4) | 87.8(80.9-94.7) | 19.8(13.2-26.4) |
| Costa Rica | 44.1(20.0-68.1) | 49.9(27.9-71.8) | 33.1(17.2-48.9) | 72.3(57.0-87.7) | 87.4(77.5-97.3) | 41(23.4-58.6) |
| Curaçao | 24.3(18.0-30.5) | 36.3(27.2-45.5) | 43.1(36.3-49.9) | 72.5(61.5-83.5) | 83.4(76.4-90.4) | 30.6(20.3-40.8) |
| El Salvador | 46.5(35.5-57.5) | 33.6(18.4-48.8) | 48.4(32.1-64.8) | 70.1(57.7-82.4) | 80.5(67.8-93.1) | 37.1(18.4-55.9) |
| Honduras | 46.6(30.2-63.0) | 32.7(16.0-49.4) | 50.2(35.4-64.9) | 82.4(77.7-87.2) | 92.2(86.0-98.5) | 42.6(31.7-53.4) |
| Jamaica | 28.2(15.8-40.6) | 42.9(31.2-54.7) | 42.5(33.7-51.2) | 53.5(45.7-61.2) | 85.5(77.8-93.2) | 27(19.1-35) |
| Peru | 36.4(29.5-43.3.0) | 28.1(21.3-34.8) | 25.2(15.7-34.7) | 82.3(70.4-94.1) | 89.8(82.5-97.1) | 26.5(18.2-34.8) |
| Saint Kitts and Nevis | 30.0 | 25.0 | 31.7 | 53.2 | 87.5 | 21.8 |
| Suriname | 36.8(29.1-44.4) | 38.7(29.8-47.6) | 42.6(32.4-52.9) | 67.8(61.7-74.0) | 79.9(70.9-89.0) | 34.8(26.6-43.0) |
| Trinidad and Tobago | 40.5(31.1-49.9) | 27.4(18.8-36) | 40(29.2-50.8) | 51.7(42.8-60.6) | 89.4(84.1-94.7) | 26.8(15.6-38.1) |
| **Western Pacific Region** | | | | | | |
| Brunei Darussalam | 14.8(8.6-20.9) | 20(12.4-27.6) | 27.7(21.5-33.9) | 68.8(61.2-76.4) | 94.4(90.6-98.2) | 53.0(44.3-61.6) |
| Cook Islands | 26.8(11.4-42.3) | 16(8.6-23.5) | 37.3(25.8-48.8) | 63.2(51.2-75.2) | 90.1(81-99.2) | 34.0(23.7-44.2) |
| Kiribati | 27.7(21.3-34.0) | 17.3(11.2-23.5) | 22.6(15.8-29.3) | 51.9(45.9-57.9) | 97.4(95.1-99.6) | 33.6(23.3-43.9) |
| Lao People's Democratic Republic | 26.1(19.8-32.3) | 12.8(6.4-19.2) | 28.8(18.9-38.8) | 96.6(91.8-100) | 88.3(81.3-95.3) | 29.5(17.3-41.7) |
| Malaysia | 13.7(11.3-16.2) | 27(23.4-30.7) | 36.1(32.3-40) | 70.7(67.3-74.2) | 95.5(94.3-96.8) | 41(36.2-45.8) |
| Mongolia | 29.8(20.9-38.7) | 24.7(18.2-31.1) | 31.8(21.2-42.4) | 78.9(74-83.8) | 87.7(81.4-94) | 23(14-32) |
| Philippines | 26.2(22.5-29.8) | 28.6(24.7-32.5) | 29.2(24.6-33.7) | 66(61.8-70.1) | 95.3(94-96.7) | 34.8(31.1-38.6) |
| Samoa | 40.2(35.1-45.3) | 35.6(31.5-39.8) | 33.5(29.9-37.2) | 47.1(39.4-54.8) | 84.5(80.2-88.7) | 36(31.3-40.8) |
| Solomon Islands | 37.6(26.4-48.8) | 35.3(30.2-40.5) | 41.1(32.1-50.2) | 38.2(24.6-51.9) | 86.6(74.6-98.6) | 26.8(12.7-40.9) |
| Tokelau | 37.5 | 18.6 | 56.2 | 69.8 | - | - |
| Tonga | 43.7(37.3-50.1) | 31.4(24.5-38.2) | 31.3(25.9-36.7) | 49.7(40.9-58.6) | 88.6(83.3-94) | 36.8(31-42.7) |
| Tuvalu | 32.5 | 22.4 | 15.3 | 77.8 | 83.7 | 32.8 |
| Vanuatu | 29(13.4-44.6) | 15.5(5.8-25.2) | 19.1(10.3-27.9) | 52.7(32.8-72.6) | 81.1(63.3-98.8) | 27.2(8.4-46.1) |
| Vietnam | 16.3(8.9-23.7) | 24.6(5.5-43.7) | 27.1(20.2-34) | 59.6(40.4-78.9) | 82(62.6-100.0) | 50.2(31.2-69.2) |
| Wallis and Futuna | 43.3(35.8-50.7) | 27.2(20.3-34.1) | 37(28.0-46.1) | 72.9(64.5-81.4) | 96.6(93.2-99.9) | 56.9(47.8-66) |
| **South-East Asia Region** | | | | | | |
| Bangladesh | 59.9(49.9-69.9) | 46.1(31.9-60.3) | 45.5(34.1-57.0) | 86.4(79.6-93.3) | 90.6(85.0-96.2) | 58.3(49.0-67.6) |
| Indonesia | 33.2(27.4-38.9) | 32.7(28.3-37.2) | 30.5(25.2-35.7) | 60.2(54-66.4) | 96.4(94.2-98.5) | 36.1(29.8-42.5) |
| Maldives | 23.1(14.8-31.5) | 23.8(17.7-29.8) | 38.3(30.7-45.9) | 59.1(50-68.2) | 89.2(84.2-94.2) | 50.7(41.7-59.6) |
| Thailand | 12.2(6.2-18.3) | 12.2(6.8-17.6) | 26.2(17.8-34.7) | 69.7(60.6-78.9) | 85.3(77.6-92.9) | 28(19.6-36.5) |
| Timor-Leste | 33.2(28.8-37.7) | 14.6(10.4-18.7) | 22.7(16.5-28.9) | 60.0(53.8-66.2) | 93.5(90.2-96.7) | 29.1(24.1-34.1) |
| **Total estimates** | 35.4(31.8-39.0) | 28.8(26.5-31.1) | 34.4(32.6-36.3) | 66.5(62.9-70.1) | 89.6(88.3-91.0) | 35.6(32.3-39.0) |
| **I^2^ (%)** | 92.8 | 82.8 | 69.8 | 92.2 | 79.7 | 90.7 |

**Supplementary Table 6**

The prevalence of individual parental and peer support among in-school normal socioeconomic status adolescents by region, and country groups (Low-income and middle-income countries, 2009–2015).

|  | **Parental support** | | | | **Peer support** | |
| --- | --- | --- | --- | --- | --- | --- |
|  | **Parents checked homework** | **Parents understood problems** | **Parents concerned free time** | **Parents respected for privacy** | **Having close friendships** | **Having supportive classmates** |
| **Africa Region** | | | | | |  |
| Benin | 48.9(45.3-52.5) | 42.3(38-46.5) | 45.8(42.4-49.3) | 76.4(73.8-78.9) | 88.6(86.2-91) | 34.8(31.3-38.3) |
| Mauritania | 49.7(45.6-53.9) | 34.1(30-38.2) | 35.8(32.4-39.3) | 69(64.6-73.4) | 93.8(92.1-95.4) | 37.9(33.8-42) |
| Mozambique | 47.5(41.3-53.7) | 47.6(40.6-54.5) | 39.8(32.8-46.8) | 80.6(75.2-86.1) | 89.9(86.5-93.3) | 35(28-42) |
| Namibia | 39(35.3-42.6) | 40.4(38-42.9) | 32.5(29.9-35) | 58.1(55.1-61.1) | 87.8(86.4-89.3) | 28.6(25.6-31.5) |
| Seychelles | 42.1(38.6-45.6) | 32.5(29.9-35.1) | 41.8(39.1-44.6) | 69.2(66.5-71.8) | 92.1(90.6-93.5) | 23.4(21-25.7) |
| Swaziland | 34.1(31.3-36.9) | 42(39.9-44.2) | 31.7(29.6-33.9) | 59.5(56.5-62.4) | 83.4(81.7-85.1) | 28.4(25.6-31.1) |
| United Republic of Tanzania | 57.1(54-60.1) | 38.6(34.6-42.6) | 38.7(34.7-42.6) | 77.2(74.8-79.7) | 90.8(89.3-92.2) | 35.8(32.1-39.5) |
| **Eastern Mediterranean Region** | | | | | | |
| Afghanistan | 44.2(36.1-52.4) | 52.7(44.9-60.5) | 54.4(47.4-61.4) | 62.9(57.9-67.9) | 88.1(85.1-91.1) | 65.2(55.9-74.5) |
| Egypt | 45(38.9-51.2) | 35.4(29.3-41.5) | 48.6(40.8-56.5) | 75.9(69.5-82.3) | 92.3(90.4-94.2) | 53.1(45.4-60.8) |
| Iraq | 48.5(44.3-52.6) | 43.1(39-47.1) | 46.8(42.2-51.4) | 87.9(85.8-90.1) | 94.6(93.4-95.7) | 49.6(45.5-53.8) |
| Kuwait | 35.4(30.9-40) | 32.3(29.2-35.4) | 42.6(38.1-47.1) | 70.3(66.4-74.2) | 92.7(90.9-94.5) | 43.7(38.4-49.1) |
| Lebanon | 44.7(40.3-49.1) | 48.1(44.4-51.8) | 53.7(50.6-56.8) | 82.8(80.2-85.4) | 96.9(95.9-97.9) | 70.7(66.5-75) |
| Morocco | 46.7(43.3-50.1) | 27.9(24.1-31.6) | 39.9(34.8-45) | 74.6(73.4-75.9) | 91.6(89.7-93.6) | 31.8(26.5-37) |
| Oman | 50.2(47.7-52.8) | 40.9(37.7-44.1) | 43.2(40.4-45.9) | 86.4(84.3-88.6) | 92.7(91.8-93.7) | 59.3(56.6-62) |
| Qatar | 33.6(29.7-37.6) | 26.3(23.3-29.3) | 27.3(23.8-30.8) | 78.5(75.9-81) | 88.1(86.1-90.2) | 44.9(39.9-49.9) |
| Syrian Arab Republic | 41.1(37.2-44.9) | 27.2(23.8-30.6) | 38.8(34.1-43.5) | 87.5(86.1-89) | 95.1(93.8-96.4) | 45.9(40.5-51.2) |
| United Arab Emirates | 46.3(42.2-50.4) | 47.5(44.3-50.7) | 50.5(47.4-53.6) | 79.1(77-81.2) | 94.1(93.1-95.2) | 67.7(63.8-71.5) |
| **America Region** | | | | | | |
| Antigua and Barbuda | 37.4(33.2-41.6) | 32.9(30.1-35.8) | 40.5(36.7-44.2) | 60.8(57.7-63.9) | 91.6(89.5-93.7) | 20.1(17.4-22.8) |
| Argentina | 30.4(29-31.9) | 49.3(47.2-51.3) | 55.2(53.4-57.1) | 68.2(66.8-69.6) | 94.5(93.7-95.3) | 51.2(49.1-53.3) |
| Barbados | 25.8(23.2-28.5) | 30.2(27.6-32.7) | 37.8(34.9-40.7) | 72.2(69.1-75.4) | 94.4(93.1-95.8) | 17.8(15.9-19.8) |
| Belize | 56.6(52.5-60.7) | 49.3(46-52.5) | 54.4(51.6-57.2) | 61.2(58.1-64.4) | 92.3(90.6-94) | 43.4(40.9-45.8) |
| Bolivia | 40.3(37.8-42.8) | 32.7(30.3-35) | 38.6(35.7-41.4) | 79(77.4-80.6) | 92.4(91.5-93.3) | 40.9(36.9-45) |
| British Virgin Islands | 35.3 | 37.7 | 48.1 | 69.0 | 91.5 | 22.6 |
| Bahamas | 50.5(47.4-53.6) | 37.6(34.1-41.1) | 46.4(42-50.8) | 64.6(61.9-67.3) | 92.5(90.4-94.6) | 25.5(21.3-29.7) |
| Costa Rica | 33.7(29.3-38.2) | 46.6(44.7-48.6) | 54.1(50.3-57.8) | 82.2(80.3-84) | 94.3(93.1-95.5) | 51.6(47.8-55.4) |
| Curaçao | 33.6(30.4-36.8) | 54.2(51.6-56.8) | 66.2(64-68.4) | 79.1(77.5-80.7) | 90.8(89.4-92.2) | 48.9(46.4-51.5) |
| El Salvador | 57(51.8-62.2) | 51.3(47.8-54.8) | 58.1(54.5-61.7) | 76.6(73.6-79.5) | 95.1(93.7-96.5) | 54.9(51.3-58.6) |
| Honduras | 53(49.2-56.8) | 48(44.1-51.9) | 56(52.5-59.5) | 81.3(78.8-83.7) | 93.7(92.3-95.1) | 45.8(43.2-48.5) |
| Jamaica | 41.5(36-46.9) | 32.5(26.6-38.4) | 39.4(34.7-44.1) | 52(45.5-58.6) | 90.4(87.6-93.2) | 22.3(18.6-25.9) |
| Peru | 43.9(41.2-46.7) | 35.4(33.1-37.8) | 36.5(33.5-39.5) | 79.9(77.4-82.4) | 94.8(94-95.7) | 43.9(41.8-46.1) |
| Saint Kitts and Nevis | 27.5 | 25.8 | 32.4 | 63.7 | 88.9 | 14.4 |
| Suriname | 37(32.2-41.8) | 42.8(40.1-45.6) | 48(44.3-51.7) | 68.7(65.3-72) | 83(80.3-85.6) | 48.6(42.9-54.4) |
| Trinidad and Tobago | 46.3(40.9-51.8) | 40.6(36.9-44.3) | 51.2(47-55.5) | 57.7(55.3-60.2) | 91.3(90.5-92.1) | 26.4(22-30.7) |
| **Western Pacific Region** | | | | | | |
| Brunei Darussalam | 14.9(13.1-16.6) | 29.1(27.1-31.1) | 42.1(39.7-44.5) | 67.1(63.9-70.4) | 97.2(96.6-97.8) | 55.9(53.4-58.3) |
| Cook Islands | 32.1(28-36.2) | 30.2(26.4-34) | 40.1(35-45.2) | 62.2(59.2-65.3) | 94(92.1-95.9) | 49.3(44.2-54.3) |
| Kiribati | 22.3(19.6-25) | 14.8(13.2-16.4) | 27.8(25-30.6) | 66.7(62.6-70.8) | 97.8(96.8-98.7) | 26.1(22.2-30) |
| Lao People's Democratic Republic | 18.9(15.3-22.6) | 16.7(15.0-18.5) | 31.3(28.5-34.0) | 91.6(90.4-92.8) | 95.4(94.3-96.4) | 19.9(17.6-22.2) |
| Malaysia | 14.2(13.4-15.0) | 31.8(30.4-33.2) | 43.6(42.4-44.9) | 74.2(73.1-75.4) | 97(96.7-97.2) | 44.6(42.9-46.4) |
| Mongolia | 47.1(45.2-48.9) | 30.6(28.6-32.7) | 49.4(47.7-51.2) | 77.6(75.7-79.4) | 94.5(93.8-95.2) | 24.8(22.8-26.7) |
| Philippines | 23.7(21.9-25.5) | 28.7(26.5-30.8) | 29.5(27.1-31.9) | 75.1(73.7-76.5) | 95.7(94.2-97.2) | 32(29.1-34.9) |
| Samoa | 42.8(39-46.6) | 30.9(27.6-34.2) | 29.6(25.4-33.7) | 54.3(49.0-59.7) | 84.2(81.1-87.4) | 33.5(29.1-37.8) |
| Solomon Islands | 36.8(32.2-41.4) | 23(18.8-27.2) | 27.7(25.5-29.8) | 40.8(35.6-46.0) | 85.8(82.6-89.0) | 26.5(22.8-30.2) |
| Tokelau | 37.0 | 23.8 | 28.4 | 63.7 | 91.9 | 32.1 |
| Tonga | 39.2(36.6-41.7) | 26(23.3-28.7) | 30.6(28.0-33.1) | 57.2(53.8-60.7) | 91.3(89.9-92.7) | 42.9(40.0-45.8) |
| Tuvalu | 28.1 | 15.8 | 18.8 | 88.9 | 85.7 | 39.9 |
| Vanuatu | 28.4(19.7-37.1) | 17.4(10.8-24.0) | 20.5(17.1-23.9) | 56.9(49.2-64.5) | 85.2(79.5-91.0) | 28.6(22.5-34.7) |
| Vietnam | 27.7(24.9-30.5) | 30.4(27.6-33.3) | 37.5(34.1-41) | 63.1(61.2-65.1) | 94.5(93.4-95.6) | 50.3(46.4-54.2) |
| Wallis and Futuna | 51.3(47.3-55.3) | 39.1(35.6-42.6) | 46.9(42.7-51.1) | 78.9(75.4-82.3) | 96.5(95.1-97.9) | 63.2(58.2-68.1) |
| **South-East Asia Region** | | | | | | |
| Bangladesh | 54.1(48.5-59.7) | 47.9(42.7-53.1) | 43.7(39.2-48.1) | 85.3(82.0-88.7) | 91.8(89.8-93.8) | 56.3(51.1-61.5) |
| Indonesia | 35.9(32.7-39.1) | 34.9(33.4-36.3) | 40.3(37.9-42.7) | 56.7(54.9-58.5) | 97.1(96.5-97.7) | 39.3(37.0-41.6) |
| Maldives | 28(25.4-30.6) | 33.1(31.4-34.9) | 47.2(44.9-49.4) | 67(64.9-69.2) | 91.3(90.1-92.5) | 60.2(57.9-62.4) |
| Thailand | 28.5(25.4-31.6) | 29.4(26.5-32.3) | 44.0(40.8-47.2) | 67.5(65.9-69.1) | 94.3(93.5-95.1) | 40.2(36.5-43.9) |
| Timor-Leste | 28.6(25.8-31.3) | 11.2(9.5-13.0) | 23.7(21.0-26.3) | 73.4(70.1-76.6) | 95.9(95.0-96.8) | 27.5(24.5-30.4) |
| **Total estimates** | 39.0(35.1-43.0) | 35.6(32.6-38.6) | 41.8(38.9-44.7) | 71.1(68.3.-73.8) | 92.6(91.8-93.4) | 41.1(37.2-45.0) |
| **I^2^ (%)** | 98.9 | 98.4 | 97.9 | 98.6 | 96.3 | 98.7 |

**Supplementary Table 7**

The prevalence of combined parental and peer support among in-school adolescents by region, and country groups (Low-income and middle-income countries, 2009–2015).

|  | Number of parental support | | | | | Number of peer support | | |
| --- | --- | --- | --- | --- | --- | --- | --- | --- |
|  | 0 | 1 | 2 | 3 | 4 | 0 | 1 | 2 |
| **Africa Region** | | | | | | | | |
| Benin | 5.1(4.2-6.0) | 28.9(26.2-31.6) | 27.6(25.0-30.1) | 23.3(21.5-25.1) | 15.1(12.6-17.7) | 8.2(6.3-10.1) | 59.8(57.1-62.5) | 32.0(28.5-35.4) |
| Mauritania | 7.6(5.6-9.6) | 37.4(33.3-41.5) | 24.1(21.1-27.1) | 22.8(19.7-25.9) | 8.1(6.4-9.9) | 4.8(3.6-5.9) | 59.2(56.2-62.1) | 36.1(32.7-39.4) |
| Mozambique | 4.3(2.5-6.2) | 30.2(24.5-35.9) | 26.1(22.8-29.4) | 23.3(20.9-25.8) | 16.1(11.0-21.1) | 7.5(4.8-10.3) | 60.6(54.8-66.4) | 31.9(26.3-37.5) |
| Namibia | 11.9(10.8-12.9) | 36.8(35.2-38.4) | 27.1(25.3-29.0) | 18.1(16.3-19.9) | 6.1(5.3-6.9) | 9.3(8.1-10.5) | 65.4(62.9-67.9) | 25.3(22.9-27.7) |
| Seychelles | 8.9(7.6-10.1) | 36.5(33.8-39.2) | 27.2(25.1-29.4) | 19.4(17.5-21.2) | 8.1(6.9-9.2) | 6.8(5.5-8.1) | 72.0(69.8-74.1) | 21.2(19.2-23.2) |
| Swaziland | 12.1(10.8-13.4) | 36.9(34.8-39.0) | 28.3(26.6-30.1) | 17.2(15.2-19.2) | 5.4(4.3-6.4) | 13.5(12.1-14.9) | 62.1(60.1-64.1) | 24.4(22.1-26.8) |
| United Republic of Tanzania | 4.3(3.3-5.3) | 31.0(28.2-33.7) | 28.7(27.1-30.2) | 22.3(20.2-24.4) | 13.7(11.5-16.0) | 7.4(6.2-8.6) | 59.3(55.8-62.7) | 33.4(29.9-36.8) |
| **Pooled estimates** | 7.8(5.1-10.4) | 34.1(31.5-36.8) | 27.4(26.4-28.4) | 20.8(18.9-22.8) | 9.8(7.5-12.2) | 8.2(6.1-10.4) | 62.8(58.8-66.7) | 29.0(24.7-33.3) |
| **I^2^ (%)** | 97.0 | 85.4 | 33.1 | 84.2 | 94.2 | 93.9 | 93.0 | 93.4 |
| **Eastern Mediterranean Region** | | | | | | | | |
| Afghanistan | 12.2(8.8-15.5) | 27.4(21.1-33.7) | 19.9(17.2-22.5) | 22.3(17.6-27.1) | 18.3(14.6-21.9) | 5.7(4.1-7.4) | 34.2(27.1-41.3) | 60.1(51.7-68.4) |
| Egypt | 8.1(3.8-12.4) | 30.7(25.3-36.2) | 25.1(21.6-28.7) | 22.3(18.1-26.6) | 13.7(9.3-18.1) | 5.5(3.7-7.2) | 45.3(38.6-52.0) | 49.2(42.0-56.5) |
| Iraq | 3.2(2.3-4.1) | 30.7(27.1-34.2) | 25.9(23.6-28.2) | 21.0(18.4-23.6) | 19.2(16.5-22.0) | 3.8(2.9-4.8) | 49.7(45.3-54.0) | 46.5(42.2-50.8) |
| Kuwait | 10.7(8.4-13.0) | 36.7(33.8-39.6) | 25.3(23.8-26.8) | 17.8(15.8-19.8) | 9.5(8.0-11.1) | 5.9(4.2-7.6) | 52.6(48.6-56.5) | 41.5(36.4-46.7) |
| Lebanon | 3.9(2.8-5.1) | 25.8(23.6-28.0) | 25.3(22.4-28.2) | 28.1(25.1-31.1) | 16.9(14.7-19.0) | 2.0(1.2-2.8) | 29.3(25.2-33.5) | 68.7(64.2-73.2) |
| Morocco | 6.8(5.3-8.2) | 38.3(33.6-43.0) | 26.6(24.3-29.0) | 18.5(16.2-20.8) | 9.8(7.5-12.0) | 7.3(5.9-8.8) | 63.5(58.9-68.1) | 29.2(24.3-34.0) |
| Oman | 3.9(3.0-4.8) | 30.3(27.9-32.8) | 26.0(24.4-27.7) | 22.4(20.2-24.5) | 17.4(15.2-19.5) | 4.9(3.9-5.9) | 39.0(36.6-41.5) | 56.1(53.6-58.6) |
| Qatar | 9.2(7.5-10.9) | 48.1(44.2-52.0) | 21.0(18.4-23.6) | 13.8(11.6-16.0) | 7.9(5.9-9.8) | 9.7(7.8-11.6) | 48.6(44.0-53.1) | 41.7(36.8-46.6) |
| Syrian Arab Republic | 4.1(2.8-5.5) | 41.3(36.6-46.0) | 25.6(22.6-28.5) | 17.9(15.5-20.4) | 11.1(9.2-12.9) | 3.8(2.5-5.1) | 53.3(48.9-57.8) | 42.9(37.9-47.8) |
| United Arab Emirates | 5.3(4.2-6.3) | 26.6(24.0-29.1) | 26.6(24.5-28.7) | 24.5(22.5-26.4) | 17.1(14.7-19.5) | 3.1(2.5-3.8) | 33.3(29.3-37.2) | 63.6(59.7-67.4) |
| **Pooled estimates** | 6.4(4.9-7.8) | 33.5(29.2-37.9) | 24.8(23.5-26.1) | 20.8(18.2-23.4) | 14.0(11.3-16.7) | 5.0(3.8-6.3) | 44.9(38.4-51.4) | 49.9(42.6-57.3) |
| **I^2^ (%)** | 90.9 | 94.0 | 69.7 | 90.5 | 92.8 | 91.1 | 95.8 | 96.2 |
| **America Region** | | | | | | | | |
| Antigua and Barbuda | 13.1(10.7-15.5) | 34.8(31.0-38.7) | 26.5(23.7-29.3) | 18.4(15.6-21.2) | 7.1(5.8-8.5) | 7.9(5.9-9.9) | 73.4(70.7-76.1) | 18.7(16.2-21.2) |
| Argentina | 9.8(8.9-10.6) | 28.8(27.5-30.2) | 24.3(23.2-25.4) | 24.8(23.5-26.0) | 12.3(11.1-13.5) | 3.9(3.3-4.6) | 47.0(45.3-48.7) | 49.1(47.1-51.0) |
| Barbados | 10.5(8.9-12.1) | 41.6(38.8-44.4) | 25.3(23.6-27.1) | 16.9(14.8-18.9) | 5.7(4.4-7.1) | 5.1(3.9-6.3) | 77.7(75.4-79.9) | 17.2(15.3-19.2) |
| Belize | 7.6(5.9-9.4) | 24.7(22.9-26.6) | 24.8(22.8-26.7) | 26.3(23.9-28.7) | 16.6(14.0-19.1) | 6.1(4.9-7.3) | 53.3(51.0-55.5) | 40.7(38.2-43.1) |
| Bolivia | 8.4(7.6-9.2) | 36.9(33.9-39.8) | 23.9(21.9-26.0) | 17.9(16.9-18.9) | 12.8(11.2-14.5) | 6.3(5.4-7.2) | 54.9(51.5-58.2) | 38.8(35.0-42.7) |
| British Virgin Islands | 9.0(7.1-10.9) | 34.5(32.5-36.5) | 27.0(25.3-28.7) | 19.5(17.3-22.7) | 9.9(7.1-12.7) | 7.4(5.4-9.4) | 71.4(69.4-73.4) | 21.2(20.0-22.4) |
| Bahamas | 8.4(6.7-10.1) | 30.4(27.1-33.8) | 28.3(25.3-31.4) | 21.9(19.8-24.0) | 10.9(8.9-12.9) | 5.8(4.3-7.3) | 71.0(67.1-74.9) | 23.2(19.0-27.3) |
| Costa Rica | 6.3(5.4-7.3) | 29.0(26.0-31.9) | 24.4(22.0-26.9) | 22.8(20.3-25.4) | 17.4(14.5-20.3) | 4.1(3.0-5.2) | 46.3(43.0-49.7) | 49.6(45.7-53.5) |
| Curaçao | 4.0(3.1-4.8) | 23.2(20.9-25.4) | 26.8(24.8-28.8) | 30.3(27.9-32.7) | 15.8(14.1-17.5) | 6.4(5.4-7.3) | 48.6(46.1-51.0) | 45.1(42.6-47.5) |
| El Salvador | 5.1(3.8-6.3) | 24.1(21.0-27.2) | 19.9(17.9-21.8) | 26.1(24.1-28.0) | 24.9(20.8-28.9) | 3.6(2.3-4.9) | 43.9(40.1-47.8) | 52.5(48.8-56.1) |
| Honduras | 4.5(3.3-5.7) | 26.5(23.4-29.6) | 20.8(19.2-22.4) | 24.2(22.5-26.0) | 24.0(20.3-27.8) | 4.6(3.4-5.8) | 51.6(49.2-54.0) | 43.8(41.4-46.2) |
| Jamaica | 12.0(9.8-14.1) | 37.7(34.1-41.2) | 28.1(24.8-31.3) | 17.7(14.5-21.0) | 4.6(2.8-6.4) | 9.0(6.1-12.0) | 69.4(63.3-75.6) | 21.5(17.3-25.8) |
| Peru | 7.0(5.8-8.1) | 36.0(33.3-38.7) | 25.5(23.8-27.2) | 18.2(16.6-19.9) | 13.3(11.7-15.0) | 4.0(3.4-4.7) | 53.9(51.7-56.0) | 42.1(40.1-44.1) |
| Saint Kitts and Nevis | 14.9(13.0-16.8) | 43.3(41.3-45.3) | 23.8(21.4-26.2) | 14.3(12.1-16.5) | 3.8(2.3-5.3) | 10.3(8.3-12.3) | 75.8(73.5-78.3) | 13.5(11.5-15.5) |
| Suriname | 7.3(6.3-8.3) | 32.7(30.1-35.3) | 27.0(24.5-29.6) | 23.3(20.9-25.8) | 9.6(7.8-11.4) | 10.8(8.4-13.2) | 48.4(44.3-52.5) | 40.8(35.5-46.1) |
| Trinidad and Tobago | 9.4(7.6-11.2) | 30.6(27.1-34.1) | 26.7(24.5-29.0) | 23.6(20.7-26.4) | 9.7(7.6-11.7) | 7.1(6.1-8.2) | 67.9(64.0-71.9) | 24.9(21.0-28.9) |
| **Pooled estimates** | 8.0(6.7-9.2) | 31.2(28.3-34.0) | 25.0(23.8-26.2) | 22.3(20.2-24.5) | 12.9(10.5-15.4) | 5.8(4.9-6.6) | 57.6(51.6-63.7) | 36.3(29.5-43.1) |
| **I^2^ (%)** | 93.5 | 94.2 | 81.3 | 94.2 | 95.9 | 87.0 | 98.5 | 98.8 |
| **Western Pacific Region** | | | | | | | | |
| Brunei Darussalam | 9.9(8.4-11.5) | 47.0(44.9-49.1) | 26.9(25.1-28.7) | 13.7(12.1-15.2) | 2.4(1.8-3.1) | 1.8(1.3-2.2) | 43.7(41.3-46.1) | 54.5(52.2-56.9) |
| Cook Islands | 13.9(10.7-17.2) | 38.9(34.9-42.9) | 25.1(21.3-28.8) | 15.3(11.7-18.9) | 6.9(4.3-9.4) | 4.8(3.3-6.2) | 49.3(45.1-53.6) | 45.9(41.5-50.3) |
| Kiribati | 14.9(12.9-16.9) | 52.6(49.8-55.4) | 21.7(19.8-23.6) | 9.0(7.6-10.4) | 1.8(1.0-2.6) | 1.6(0.8-2.3) | 72.2(68.4-76.1) | 26.2(22.4-30.0) |
| Lao People's Democratic Republic | 4.9(4.0-5.9) | 51.8(49.0-54.6) | 27.2(25.2-29.1) | 11.8(10.4-13.3) | 4.2(2.9-5.5) | 4.1(3.2-5.0) | 76.7(74.2-79.2) | 19.2(16.9-21.5) |
| Malaysia | 8.1(7.5-8.7) | 43.9(42.8-45.0) | 28.7(27.9-29.6) | 15.6(14.8-16.4) | 3.7(3.4-4.1) | 2.3(2.1-2.6) | 54.0(52.2-55.7) | 43.7(42.0-45.4) |
| Mongolia | 7.4(6.6-8.3) | 31.1(29.4-32.8) | 26.0(24.5-27.4) | 21.2(20.0-22.4) | 14.3(13.0-15.6) | 4.7(4.0-5.4) | 71.5(69.6-73.3) | 23.8(22.0-25.7) |
| Philippines | 11.4(10.4-12.5) | 46.6(44.5-48.8) | 22.4(20.4-24.4) | 13.1(11.8-14.4) | 6.5(5.6-7.3) | 3.8(2.4-5.3) | 64.4(62.7-66.2) | 31.7(29.1-34.3) |
| Samoa | 14.0(11.6-16.3) | 39.5(36.5-42.5) | 26.5(23.1-29.9) | 15.8(13.8-17.8) | 4.2(2.4-6.0) | 11.1(8.5-13.8) | 59.0(55.7-62.3) | 29.8(26.1-33.6) |
| Solomon Islands | 22.1(18.9-25.3) | 40.6(37.2-43.9) | 22.9(18.8-27.0) | 12.4(9.6-15.2) | 2.0(0.8-3.2) | 11.8(8.9-14.6) | 63.9(59.5-68.3) | 24.3(20.4-28.2) |
| Tokelau | 19.5(18.1-20.9) | 32.7(31.5-33.9) | 27.6(25.6-29.7) | 14.1(12.9-15.3) | 6.1(4.8-7.4) | 6.7(5.2-8.2) | 62.6(61.0-64.2) | 30.7(29.0-32.4) |
| Tonga | 15.1(12.8-17.4) | 38.6(36.3-40.9) | 27.6(25.6-29.6) | 15.3(13.6-17.0) | 3.3(2.2-4.4) | 6.6(5.5-7.7) | 54.0(51.3-56.6) | 39.4(36.6-42.2) |
| Tuvalu | 4.2(2.2-6.2) | 57.8(55.2-60.4) | 25.5(23.0-27.9) | 8.3(6.1-10.5) | 4.3(3.5-5.1) | 8.1(7.2-10.0) | 58.9(57.0-60.8) | 33.0(31.4-34.6) |
| Vanuatu | 23.6(19.4-27.7) | 42.6(38.4-46.9) | 22.7(18.5-26.9) | 9.8(5.1-14.6) | 1.3(0.0-2.6) | 10.3(6.1-14.5) | 65.6(59.1-72.1) | 24.1(18.4-29.8) |
| Vietnam | 15.9(14.4-17.5) | 38.8(36.0-41.6) | 22.3(20.4-24.2) | 16.5(14.6-18.4) | 6.5(5.1-7.8) | 3.8(2.9-4.6) | 47.8(43.8-51.8) | 48.5(44.7-52.2) |
| Wallis and Futuna | 5.4(3.9-6.9) | 28.0(24.7-31.3) | 29.4(26.5-32.2) | 24.0(21.2-26.7) | 13.3(11.1-15.4) | 1.9(1.1-2.8) | 37.8(33.3-42.3) | 60.2(55.6-64.9) |
| **Pooled estimates** | 12.5(10.3-14.7) | 41.6(37.8-45.3) | 25.4(23.8-27.0) | 14.9(12.9-17.0) | 5.3(3.7-6.9) | 4.6(3.6-5.6) | 58.5(52.3-64.6) | 36.2(29.2-43.3) |
| **I^2^ (%)** | 97.1 | 97.0 | 87.8 | 95.3 | 97.3 | 94.8 | 98.5 | 98.7 |
| **South-East Asia Region** | | | | | | | | |
| Bangladesh | 3.4(1.7-5.1) | 22.9(18.7-27.1) | 31.0(26.6-35.5) | 23.8(20.6-26.9) | 18.9(15.1-22.7) | 5.1(3.6-6.6) | 41.3(37.4-45.3) | 53.5(49.2-57.9) |
| Indonesia | 11.9(10.9-13.0) | 36.1(34.5-37.6) | 29.5(28.1-31.0) | 17.6(16.8-18.5) | 4.8(4.3-5.4) | 2.2(1.7-2.6) | 59.4(57.2-61.6) | 38.4(36.1-40.6) |
| Maldives | 11.5(10.2-12.8) | 36.8(34.8-38.8) | 25.7(23.9-27.5) | 18.5(16.6-20.4) | 7.5(6.3-8.7) | 5.8(4.8-6.8) | 37.7(35.6-39.8) | 56.5(54.4-58.7) |
| Thailand | 11.0(9.8-12.3) | 38.3(35.5-41.1) | 27.8(26.3-29.3) | 17.8(15.6-20.0) | 5.1(3.6-6.6) | 4.1(3.2-4.9) | 58.2(55.3-61.2) | 37.7(34.4-41.0) |
| Timor-Leste | 11.8(10.2-13.3) | 52.5(50.2-54.7) | 25.4(23.4-27.3) | 8.7(7.6-9.8) | 1.7(1.1-2.3) | 3.6(2.8-4.5) | 69.5(66.6-72.3) | 26.9(24.1-29.7) |
| **Pooled estimates** | 10.0(7.3-12.6) | 37.4(30.0-44.9) | 27.6(25.7-29.4) | 17.2(12.3-22.0) | 6.9(4.2-9.6) | 4.1(2.7-5.5) | 53.2(41.2-65.3) | 42.6(31.4-53.8) |
| **I^2^ (%)** | 95.0 | 98.1 | 77.9 | 98.1 | 97.5 | 92.9 | 99.0 | 98.8 |
| **Total** |  |  |  |  |  |  |  |  |
| **Pooled estimates** | 8.8(6.8-10.8) | 35.3(31.6-39.1) | 26.0(24.8-27.2) | 19.3(16.3-22.3) | 9.7(6.2-13.2) | 5.3(4.3-6.3) | 55.8(49.2-62.4) | 38.4(30.7-46.0) |
| **I^2^ (%)** | 82.2 | 79.8 | 76.2 | 86.4 | 91.3 | 69.7 | 81.7 | 84.1 |

**Supplementary Table 8**

The prevalence of combined parental and peer support among in-school adolescents by region, country income level (Low-income and middle-income countries, 2009–2015).

|  | **Number of parental support** | | | | | **Number of peer support** | | |
| --- | --- | --- | --- | --- | --- | --- | --- | --- |
|  | **0** | **1** | **2** | **3** | **4** | **0** | **1** | **2** |
| **Global** | 8.8(6.8-10.8) | 35.3(31.6-39.1) | 26.0(24.8-27.2) | 19.3(16.3-22.3) | 9.7(6.2-13.2) | 5.3(4.3-6.3) | 55.8(49.2-62.4) | 38.4(30.7-46.0) |
| **Regions** | | | | | | | | |
| Africa Region | 7.8(5.1-10.4) | 34.1(31.5-36.8) | 27.4(26.4-28.4) | 20.8(18.9-22.8) | 9.8(7.5-12.2) | 8.2(6.1-10.4) | 62.8(58.8-66.7) | 29.0(24.7-33.3) |
| Eastern Mediterranean Region | 6.4(4.9-7.8) | 33.5(29.2-37.9) | 24.8(23.5-26.1) | 20.8(18.2-23.4) | 14.0(11.3-16.7) | 5.0(3.8-6.3) | 44.9(38.4-51.4) | 49.9(42.6-57.3) |
| America Region | 8.0(6.7-9.2) | 31.2(28.3-34.0) | 25.0(23.8-26.2) | 22.3(20.2-24.5) | 12.9(10.5-15.4) | 5.8(4.9-6.6) | 57.6(51.6-63.7) | 36.3(29.5-43.1) |
| Western Pacific Region | 12.5(10.3-14.7) | 41.6(37.8-45.3) | 25.4(23.8-27.0) | 14.9(12.9-17.0) | 5.3(3.7-6.9) | 4.6(3.6-5.6) | 58.5(52.3-64.6) | 36.2(29.2-43.3) |
| South-East Asia Region | 10.0(7.3-12.6) | 37.4(30.0-44.9) | 27.6(25.7-29.4) | 17.2(12.3-22.0) | 6.9(4.2-9.6) | 4.1(2.7-5.5) | 53.2(41.2-65.3) | 42.6(31.4-53.8) |
| **Country income level** | | | | | | | | |
| Low | 5.8(3.9-7.6) | 29.8(28.0-31.5) | 25.6(21.7-29.6) | 22.9(21.8-24.1) | 15.4(13.4-17.3) | 7.1(6.1-8.2) | 54.0(45.7-62.3) | 38.5(30.1-46.9) |
| Lower middle | 10.4(8.7-120) | 37.6(34.0-41.2) | 25.0(23.7-26.2) | 17.6(15.4-19.7) | 8.8(7.1-10.6) | 5.7(4.6-6.7) | 57.8(53.2-62.3) | 36.3(31.4-41.2) |
| Upper middle | 8.4(6.7-10.1) | 34.1(29.9-38.2) | 26.4(25.2-27.6) | 20.5(17.9-23.0) | 10.4(7.6-13.1) | 5.2(4.0-6.5) | 54.1(48.0-60.1) | 40.3(32.9-47.7) |
| High | 7.9(6.2-9.7) | 35.1(29.6-40.6) | 26.0(25.0-27.0) | 20.4(17.2-23.5) | 10.4(7.0-13.8) | 5.6(4.1-7.1) | 55.5(45.2-65.7) | 38.9(27.7-50.1) |

**Supplementary Table 9** The relationship between the number of parental and peer support with mental distress and health risk behaviours in male in-school adolescents (Low-income and middle-income countries, 2009–2015).

|  | **Loneliness** | **Insomnia due to anxiety** | **Suicidal ideation** | **Suicidal plan** | **Suicidal attempt** | **Violence** | **Hygiene practices** | **Premature sexual** | **Tobacco use** | **Alcohol**  **use** | **Marijuana**  **use** | **Sedentary** | **School truancy** |
| --- | --- | --- | --- | --- | --- | --- | --- | --- | --- | --- | --- | --- | --- |
| Number of parental support | | | | | |  |  |  |  |  |  |  |  |
| 0 | 1 [Reference] | 1  [Reference] | 1 [Reference] | 1 [Reference] | 1 [Reference] | 1 [Reference] | 1 [Reference] | 1 [Reference] | 1 [Reference] | 1 [Reference] | 1 [Reference] | 1 [Reference] | 1 [Reference] |
| 1 | 0.77  (0.71-0.84) | 0.78  (0.71-0.86) | 0.73  (0.68-0.79) | 0.79  (0.72-0.85) | 0.69  (0.64-0.75) | 0.69  (0.65-0.73) | 1.00  (0.95-1.06) | 0.88  (0.76-1.02) | 0.74  (0.68-0.8) | 0.76  (0.71-0.81) | 0.61  (0.55-0.69) | 0.87  (0.83-0.93) | 0.75  (0.71-0.79) |
| 2 | 0.73  (0.67-0.8) | 0.74  (0.67-0.82) | 0.58  (0.53-0.63) | 0.63  (0.58-0.69) | 0.56  (0.51-0.61) | 0.63  (0.59-0.67) | 0.81  (0.77-0.86) | 0.72  (0.61-0.84) | 0.58  (0.53-0.64) | 0.67  (0.62-0.72) | 0.45  (0.4-0.51) | 0.98  (0.92-1.04) | 0.61  (0.58-0.65) |
| 3 | 0.61  (0.55-0.67) | 0.68  (0.6-0.75) | 0.51  (0.47-0.56) | 0.56  (0.5-0.61) | 0.49  (0.44-0.54) | 0.53  (0.5-0.57) | 0.69  (0.65-0.74) | 0.74  (0.63-0.88) | 0.38  (0.34-0.42) | 0.61  (0.57-0.66) | 0.37  (0.32-0.43) | 0.9  (0.84-0.96) | 0.48  (0.45-0.52) |
| 4 | 0.54  (0.47-0.61) | 0.55  (0.48-0.64) | 0.39  (0.35-0.45) | 0.49  (0.43-0.55) | 0.39  (0.34-0.45) | 0.43  (0.4-0.47) | 0.60  (0.55-0.64) | 0.72  (0.59-0.88) | 0.26  (0.23-0.31) | 0.52  (0.47-0.58) | 0.21  (0.16-0.27) | 0.68  (0.63-0.73) | 0.37  (0.34-0.41) |
| Number of peer support | | | | | |  |  |  |  |  |  |  |  |
| 0 | 1 [Reference] | 1  [Reference] | 1 [Reference] | 1 [Reference] | 1 [Reference] | 1 [Reference] | 1 [Reference] | 1 [Reference] | 1 [Reference] | 1 [Reference] | 1 [Reference] | 1 [Reference] | 1 [Reference] |
| 1 | 0.48  (0.44-0.53) | 0.64  (0.58-0.72) | 0.46  (0.42-0.51) | 0.47  (0.42-0.51) | 0.45  (0.41-0.5) | 0.88  (0.81-0.95) | 0.98  (0.91-1.05) | 0.74  (0.61-0.9) | 0.68  (0.61-0.76) | 0.98  (0.89-1.08) | 0.60  (0.51-0.69) | 1.10  (1.01-1.18) | 1.05  (0.97-1.13) |
| 2 | 0.47  (0.42-0.52) | 0.66  (0.59-0.74) | 0.37  (0.33-0.41) | 0.38  (0.34-0.42) | 0.35  (0.32-0.39) | 0.71  (0.65-0.76) | 0.87  (0.81-0.94) | 0.56  (0.46-0.69) | 0.59  (0.53-0.66) | 1.03  (0.93-1.13) | 0.56  (0.48-0.66) | 1.29  (1.19-1.39) | 0.95  (0.88-1.02) |

**Supplementary Table 10** The relationship between the number of parental and peer support with mental distress and health risk behaviours in female in-school adolescents (Low-income and middle-income countries, 2009–2015).

|  | **Loneliness** | **Insomnia due to anxiety** | **Suicidal ideation** | **Suicidal plan** | **Suicidal attempt** | **Violence** | **Hygiene practices** | **Premature sexual** | **Tobacco use** | **Alcohol**  **use** | **Marijuana**  **use** | **Sedentary** | **School truancy** |
| --- | --- | --- | --- | --- | --- | --- | --- | --- | --- | --- | --- | --- | --- |
| Number of parental support | | | | | |  |  |  |  |  |  |  |  |
| 0 | 1 [Reference] | 1  [Reference] | 1 [Reference] | 1 [Reference] | 1 [Reference] | 1 [Reference] | 1 [Reference] | 1 [Reference] | 1 [Reference] | 1 [Reference] | 1 [Reference] | 1 [Reference] | 1 [Reference] |
| 1 | 0.74  (0.69-0.79) | 0.70  (0.65-0.75) | 0.63  (0.59-0.67) | 0.69  (0.64-0.73) | 0.66  (0.62-0.71) | 0.67  (0.63-0.7) | 1.06  (1.00-1.12) | 1.21  (1-1.45) | 0.66  (0.6-0.74) | 0.71  (0.67-0.76) | 0.61  (0.53-0.72) | 0.84  (0.79-0.88) | 0.82  (0.78-0.87) |
| 2 | 0.62  (0.58-0.66) | 0.61  (0.57-0.66) | 0.44  (0.42-0.48) | 0.51  (0.48-0.55) | 0.5  (0.46-0.53) | 0.55  (0.52-0.58) | 0.97  (0.91-1.02) | 0.93  (0.76-1.13) | 0.47  (0.42-0.52) | 0.6  (0.56-0.65) | 0.48  (0.41-0.57) | 0.85  (0.80-0.90) | 0.63  (0.6-0.67) |
| 3 | 0.48  (0.45-0.53) | 0.54  (0.49-0.59) | 0.36  (0.33-0.38) | 0.42  (0.39-0.46) | 0.41  (0.38-0.45) | 0.47  (0.44-0.5) | 0.84  (0.79-0.89) | 0.83  (0.67-1.03) | 0.34  (0.29-0.39) | 0.58  (0.54-0.63) | 0.4  (0.33-0.49) | 0.77  (0.73-0.82) | 0.47  (0.44-0.5) |
| 4 | 0.4  (0.36-0.44) | 0.49  (0.44-0.54) | 0.25  (0.22-0.28) | 0.31  (0.28-0.35) | 0.31  (0.28-0.35) | 0.38  (0.35-0.41) | 0.82  (0.77-0.88) | 0.77  (0.58-1.03) | 0.20  (0.16-0.25) | 0.43  (0.39-0.48) | 0.16  (0.12-0.23) | 0.55  (0.51-0.59) | 0.35  (0.33-0.38) |
| Number of peer support | | | | | |  |  |  |  |  |  |  |  |
| 0 | 1 [Reference] | 1  [Reference] | 1 [Reference] | 1 [Reference] | 1 [Reference] | 1 [Reference] | 1 [Reference] | 1 [Reference] | 1 [Reference] | 1 [Reference] | 1 [Reference] | 1 [Reference] | 1 [Reference] |
| 1 | 0.46  (0.42-0.5) | 0.64  (0.58-0.7) | 0.59  (0.55-0.65) | 0.55  (0.51-0.6) | 0.50  (0.46-0.55) | 0.75  (0.7-0.81) | 1.21  (1.12-1.3) | 0.70  (0.54-0.9) | 0.63  (0.55-0.73) | 0.99  (0.9-1.1) | 0.69  (0.55-0.85) | 1.15  (1.06-1.24) | 1.02  (0.94-1.1) |
| 2 | 0.38  (0.35-0.42) | 0.63  (0.57-0.69) | 0.47  (0.43-0.51) | 0.43  (0.39-0.47) | 0.37  (0.34-0.41) | 0.56  (0.52-0.6) | 0.99  (0.92-1.06) | 0.53  (0.41-0.69) | 0.49  (0.42-0.57) | 0.9  (0.81-1) | 0.56  (0.44-0.7) | 1.41  (1.3-1.52) | 0.95  (0.88-1.03) |

**Supplementary Table 11** The relationship between the number of parental or peer support with mental distress and health risk behaviours in no peer support or no parental support adolescents (Low-income and middle-income countries, 2009–2015).

|  | **Loneliness** | **Insomnia due to anxiety** | **Suicidal ideation** | **Suicidal plan** | **Suicidal attempt** | **Violence** | **Hygiene practices** | **Premature sexual** | **Tobacco use** | **Alcohol**  **use** | **Marijuana**  **use** | **Sedentary** | **School truancy** |
| --- | --- | --- | --- | --- | --- | --- | --- | --- | --- | --- | --- | --- | --- |
| Number of parental support in no peer support adolescents | | | | | |  |  |  |  |  |  |  |  |
| 0 | 1 [Reference] | 1  [Reference] | 1 [Reference] | 1 [Reference] | 1 [Reference] | 1 [Reference] | 1 [Reference] | 1 [Reference] | 1 [Reference] | 1 [Reference] | 1 [Reference] | 1 [Reference] | 1 [Reference] |
| 1 | 0.72  (0.6-0.86) | 0.77  (0.63-0.96) | 0.80  (0.67-0.95) | 0.92  (0.76-1.11) | 0.81  (0.67-0.97) | 0.64  (0.54-0.76) | 1.23  (1.05-1.44) | 0.89  (0.56-1.41) | 0.71  (0.57-0.89) | 0.85  (0.7-1.05) | 0.80  (0.58-1.09) | 0.67  (0.56-0.79) | 0.69  (0.59-0.81) |
| 2 | 0.69  (0.57-0.84) | 0.77  (0.61-0.97) | 0.60  (0.49-0.74) | 0.65  (0.52-0.81) | 0.66  (0.53-0.81) | 0.56  (0.47-0.68) | 0.88  (0.74-1.05) | 0.72  (0.43-1.19) | 0.43  (0.33-0.57) | 0.73  (0.58-0.92) | 0.46  (0.31-0.69) | 0.80  (0.67-0.97) | 0.55  (0.46-0.65) |
| 3 | 0.52  (0.41-0.66) | 0.64  (0.49-0.85) | 0.46  (0.36-0.58) | 0.55  (0.43-0.71) | 0.57  (0.45-0.73) | 0.48  (0.39-0.59) | 0.71  (0.58-0.87) | 0.67  (0.37-1.2) | 0.31  (0.22-0.44) | 0.62  (0.47-0.81) | 0.25  (0.14-0.44) | 0.69  (0.56-0.85) | 0.40  (0.33-0.5) |
| 4 | 0.60  (0.44-0.83) | 0.55  (0.37-0.83) | 0.39  (0.28-0.56) | 0.36  (0.24-0.53) | 0.38  (0.27-0.55) | 0.34  (0.26-0.44) | 0.49  (0.36-0.66) | 0.86  (0.34-2.21) | 0.25  (0.14-0.44) | 0.47  (0.31-0.7) | 0.23  (0.09-0.57) | 0.54  (0.4-0.73) | 0.27  (0.19-0.37) |
| Number of peer support in no parental support adolescents | | | | | |  |  |  |  |  |  |  |  |
| 0 | 1 [Reference] | 1  [Reference] | 1 [Reference] | 1 [Reference] | 1 [Reference] | 1 [Reference] | 1 [Reference] | 1 [Reference] | 1 [Reference] | 1 [Reference] | 1 [Reference] | 1 [Reference] | 1 [Reference] |
| 1 | 0.46  (0.39-0.54) | 0.67  (0.55-0.82) | 0.59  (0.5-0.69) | 0.58  (0.48-0.69) | 0.56  (0.47-0.66) | 0.76  (0.64-0.89) | 1.20  (1.03-1.38) | 0.68  (0.44-1.05) | 0.65  (0.53-0.79) | 1.08  (0.9-1.31) | 0.74  (0.55-0.98) | 0.91  (0.78-1.06) | 0.91  (0.79-1.06) |
| 2 | 0.44  (0.36-0.52) | 0.73  (0.59-0.9) | 0.53  (0.44-0.64) | 0.54  (0.44-0.65) | 0.47  (0.39-0.56) | 0.6  (0.51-0.71) | 0.9  (0.77-1.06) | 0.52  (0.33-0.83) | 0.51  (0.41-0.64) | 1.13  (0.93-1.38) | 0.60  (0.44-0.83) | 1.19  (1.01-1.4) | 0.86  (0.73-1) |
